# Bioelectrical Impedance Analysis in Professional and Semi-Professional Football: A Scoping Review

**DOI:** 10.3390/sports13100348

**Published:** 2025-10-03

**Authors:** Íñigo M. Pérez-Castillo, Alberto Valiño-Marques, José López-Chicharro, Felipe Segura-Ortiz, Ricardo Rueda, Hakim Bouzamondo

**Affiliations:** 1 Research and Development, Abbott Nutrition, 68 Camino de Purchil, 18004 Granada, Spain; ricardo.rueda@abbott.com; 2 Medical Services, Real Madrid, 28055 Madrid, Spain; avalino@realmadrid.es (A.V.-M.); jlchicharro@ext.realmadrid.es (J.L.-C.); fsegura@realmadrid.es (F.S.-O.); 3 Research and Development, Abbott Nutrition, Chicago, IL 60064, USA; hakim.bouzamondo@abbott.com

**Keywords:** BIA, BIVA, football, soccer, body composition, injury, phase angle

## Abstract

Background: Bioelectrical impedance analysis (BIA) is a widely used field technique for assessing body composition in football. However, its reliance on population-specific regression equations limits its accuracy. Objective: This scoping review aimed to map the scientific literature on BIA applications in professional and semi-professional football, highlighting uses, limitations, and research opportunities. Methods: A comprehensive search was conducted in the scientific databases PubMed, EMBASE, Web of Science, and SPORTDiscus. Identified studies involved the use of BIA in professional and semi-professional football players (≥16 years) in the context of routine training and competition. Results: From 14,624 records, 39 studies met the inclusion criteria and were included. Three main applications were identified: (1) quantitative body composition assessment, (2) qualitative/semi-quantitative analysis (e.g., bioelectrical impedance vector analysis (BIVA)), and (3) muscle health and injury monitoring. Seven specific research areas emerged, including hydration monitoring, cross-method validation of body composition analyses, development of predictive models, sport phenotype identification, tracking training adaptations, performance/load assessment via phase angle, and localized BIA for injury diagnosis and recovery. Conclusions: While quantitative BIA estimates may lack individual-level precision, raw parameter analyses may offer valuable insights into hydration, cellular integrity, and muscle injury status, yet further research is needed to fully realize these applications.

## 1. Introduction

Optimizing performance, preventing injuries, and ensuring a successful return to training and competition are arguably the most pursued goals in sports science. All these are aspects influenced by the athlete’s body composition, thereby explaining the great interest that body composition monitoring has garnered among sports professionals throughout the years. Current techniques for estimating body composition in sports settings typically aim to quantitatively characterize the human body into different compartments, most notably fat mass (FM), fat-free mass (FFM) and, in some cases, bone mass, whereas more comprehensive or accurate methodologies are primarily reserved for research purposes (e.g., magnetic resonance imaging (MRI)) [1]. Common techniques for body composition analysis in field settings include anthropometric measurements, dual-energy X-ray absorptiometry (DXA), and bioelectrical impedance analysis (BIA) [1]. Arguably, anthropometry is the most widely used approach for body composition assessment in sports nutrition following international standardized protocols, yet a clear limitation is the necessity for a qualified anthropometrist, coupled with the time-intensive nature of the procedure [2]. DXA is often used as a reference method because it not only estimates FM and FFM but also differentiates FFM into bone mineral content and lean soft tissue [3]. Additionally, DXA assessments are fast, relatively accurate and reliable, and do not heavily depend on water fluctuations, making them convenient for data collection in athletes [4]. However, the need for low doses of radiation, lack of portability, and requirement for a trained technician are disadvantages that have driven interest for simpler, alternative methods for repeated field assessments [3].

BIA has gained attention as a low-cost and more accessible alternative method for assessing body composition in recent years [5]. Notably, the need for validated equations along with different limitations of the technique (e.g., inter-device differences, impact of base hydration status, acute effects of exercise, etc.) compromises the accuracy of quantitative estimations in individual athletes [6]. Despite these limitations, an increasing number of studies report BIA-derived estimations of body composition outcomes to validate the efficacy of interventions, among different purposes, without considering the accuracy of such analyses or providing comparisons with different methods. Perhaps more important, BIA provides insights into raw electrical parameters of the human body, which can be directly applied to conduct semi-quantitative analyses of body composition outcomes without having to rely on the above-mentioned mathematical models, thereby offering new applications in sports medicine and nutrition [7]. How these novel applications can be leveraged by sports professional to support athlete monitoring protocols remains an area of active scientific research.

Football (soccer) is an intermittent sport with distinct seasonal phases of training and non-training periods. Body composition analysis is important to guide sports professionals in the prescription of nutritional and training protocols aimed at ensuring that professional and semi-professional players meet physical fitness requirements of competition. Detraining periods, including off-season (transition) and injury recovery, can reverse training adaptations and negatively impact fitness level and performance upon returning to competition [8]. Body composition assessment can be particularly important during pre-season—a relatively short and intensive training period—to help evaluate baseline fitness status, monitor training adaptations, and potentially prevent injuries in-season [9,10]. Further, the physical demands faced by professional football players during matches have steeply increased throughout recent years [11], and the evaluation of post-match recovery of muscle function remains a key topic in the current literature [12]. Optimizing body composition following healthy and safe recommendations is key to meeting these demands. Tailoring these strategies to each player’s position can provide a competitive advantage, which may be exploited for tactical purposes [13].

Whole body BIA, as a safe, accessible, and user-friendly methodology, has been proposed to support the monitoring of body composition through the use of predictive equations specific to professional football players and validated against accurate methodologies [14]. Alternatively, sports professionals have explored segmental/regional BIA to estimate lower body composition outcomes with equations validated for athletic populations [15], which is proposed to be particularly informative in football, where performance is often measured as sprinting and jumping ability [16]. However, the use of quantitative estimations to inform routine assessments has been severely criticized due to the above-mentioned limitations [7]. Besides estimations of FM and FFM (among different compartments), novel applications of BIA such as bioimpedance vector analysis (BIVA) enable comparisons of bioelectrical parameters and mean confidence limits or percentages with reference values, corrected by height or cross-sectional area, that can be applied without dependency on population-specific equations [17], and provide semi-quantitative insights into hydration and body cell mass (BCM). This technique might yield information to characterize football players in cross-sectional analyses or help to evaluate short-term or long-term changes in response to intensive training periods (i.e., pre-season) [18,19]. Last, localized BIA may be sensitive to changes in cell membrane integrity and edema in localized muscles, a characteristic that some authors have utilized to facilitate the assessment and monitoring of football-related injuries [20].

Previous studies have endeavored to explore recent applications of BIA/BIVA in different sports settings [21]; however, interpretations of bioelectrical impedance measurements require equations and tolerance/confidence ellipses that may be sport- and even position-specific [14,22]. Further, the features of body composition that are associated with performance, recovery, injury, and return to play, are also sport-dependent [7]. Despite the diverse applications of BIA in sports, its utilization in professional football remains ambiguous. Sports professionals and scientists face challenges in effectively leveraging this technique, understanding its limitations, and identifying existing research gaps. For this purpose, we aimed to systematically map the scientific evidence on the applications and research areas of the bioelectrical impedance technique to the monitoring of professional and semi-professional football players using a scoping review approach in order to guide researchers and practitioners and provide a better understanding of the technique in this sport discipline.

The theoretical basis of bioelectrical impedance requires an understanding of biophysical concepts that are beyond the scope of this work and have been discussed by other authors [23]. In Box 1, simple explanations of key concepts necessary for the understanding of bioelectrical impedance analyses are provided.

Box 1 **Concepts of bioelectrical impedance analysis.**

**Figure 1:**
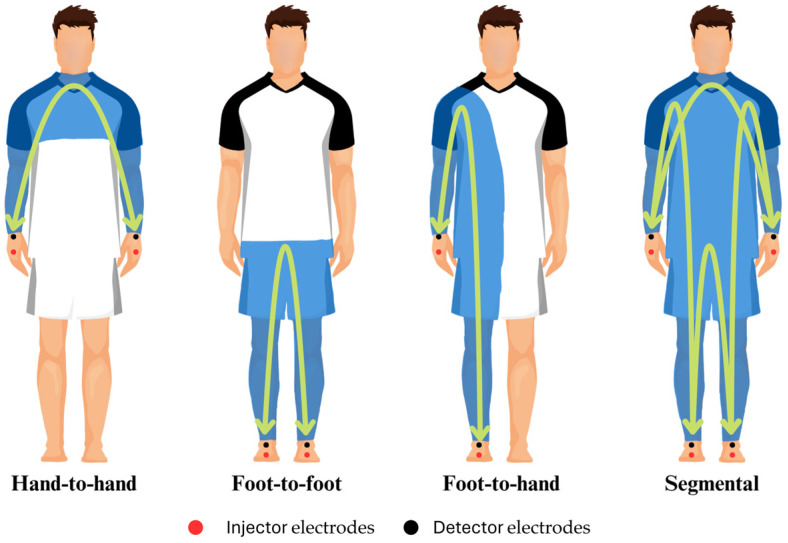
Classic configurations in bioelectrical impedance analysis.

  When an alternating current flows through a biological tissue, the matter opposes the movement of electrons, and the opposition of the matter to the applied voltage (voltage/current) is termed resistance (R). However, the electric current is also temporarily retained by cell membranes and interfaces, to be later released into the system. This retention and subsequent release lead to a delay in the current phase, which is dependent on the frequency (f) of the voltage applied, and is termed capacitive reactance (Xc). The opposition of biological tissues to an alternating current is therefore a factor of R and Xc and is a mathematically complex number termed bioimpedance (Z). An additional parameter, the phase angle (PhA), consists of the angle between the applied voltage and the resulting current, and is mathematically calculated in degrees as ArcTan Xc/R · 180°/π [23]. Phase-sensitive classic single-frequency BIA (SF-BIA) devices are based on the introduction of a low-level constant alternating sinusoidal current (≈50 kHz) through tetrapolar surface electrodes placed in different configurations (Figure 1) to determine PhA and Z. It should be noted that only phase-sensitive devices allow for the calculation of Xc from PhA determinations (X_c_ = Z · sin PhA).

  These measurements can be mathematically modeled as series-equivalent or parallel equivalent models to calculate R and Xc, with commercial devices and most available research predominantly reporting series-equivalent models [23]. While these are extensive electrical properties (size-dependent), intrinsic components of the tissue (non-size-dependent) namely resistivity and permittivity must be considered because not all tissues conduct electricity equally. At the low-level frequencies typically used in SF-BIA, the current flows mainly through extracellular fluid (the largely resistive element), and is delayed by cell membrane interfaces (the largely capacitive component). In a single-frequency series-equivalent model, R strongly correlates with Z and total body water (TBW), while Xc is considered as a semi-quantitative index of body cell content (i.e., “cells in water”). PhA is typically interpreted as an index of membrane integrity and directly correlates with the intracellular/extracellular water (ICW:ECW) ratio. Since TBW is directly correlated with R, following Ohm’s Law basis and using regression analysis, TBW can be estimated assuming that the human body is a cylinder of constant geometry and composition, using dilution methods as reference measurement [24]. Early studies leveraged these principles to estimate fat-free mass (FFM) from total body water (TBW), assuming a constant hydration index of 73%, and, consequently, fat mass (FM) was determined by subtracting FFM from total body weight. It is also important to clarify that BIA is a double-indirect method and bioimpedance measurements are impacted by fluid and electrolytes; thus, non-conductive tissues such as a FM cannot be directly quantified but are estimated instead. Through validation with gold-standard methods for each different compartment, several BIA devices use regression equations to estimate different outcomes to those already mentioned (e.g., muscle mass and bone mineral content). Naturally, the human body does not perfectly meet the assumptions of Ohm’s Law (limbs and geometrical differences, tissue compositions, etc.) and the FFM constant hydration percentage is actually variable, thus making it critical to develop and validate multiple regression equations for specific populations, which is key when interpreting BIA outcomes [23]. For example, a FFM hydration percentage ranging from 69% to 75% has been reported in healthy athletes of different sport disciplines, which might be influenced by factors such as muscle glycogen content and age [25]. Details on the validity of these models have been extensively discussed elsewhere [26].

  Multi-frequency bioimpedance (MF-BIA) and bioimpedance spectroscopy (BIS) assume that at low frequencies (e.g., 5 kHz or below), the current does not effectively penetrate cell membranes and travels largely through ECW, while at high frequencies, the current completely penetrates all body structures, thus strongly correlating with TBW. On this matter, assumptions of such frequency-based dichotomy have been subjected to criticism. Lukaski et al. affirm that, even at the lowest frequencies, a portion of the current may penetrate cell membranes, particularly when aligned with muscle fiber orientation [27]. MF-BIA has been proposed to report more accurate predictions of ECW and TBW through multiple-frequency impedance (e.g., <10 kHz for ECW and 100 kHz for TBW) using linear regression [28]. Contrary to SF-BIA and MF-BIA, phase-sensitive BIS devices use a range of frequencies (e.g., 5–1000 kHz) to measure PhA, Z, and Xc, applying non-linear least-square modeling, and then use Cole plot mathematical models to plot R_0_ (considered as resistance of ECW) and R_∞_ (considered as resistance of TBW). These in turn are used in different mathematical models to separately estimate ECW and, by subtraction, ICW. It should be noted that the assumptions underlying BIS impedance modeling, such as constant body geometry and resistivity of ECW, may not be valid in athletic populations. Variations in regional girths and anisotropic tissue properties might affect the accuracy of BIS-derived estimates in athletes [29]. Ultimately, the differences between all these above-mentioned estimations lay in the mathematical approaches followed, and whether MF-BIA or BIS are more accurate than SF-BIA to estimate body composition outcomes is debatable [27].

**Figure 2:**
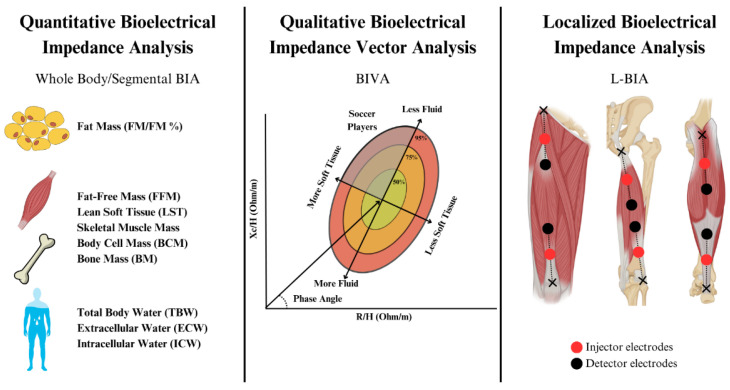
Summary of bioelectrical impedance analysis modalities.

  The above-mentioned approaches require the use of predictive equations to quantitatively estimate body composition, which can lead to errors due to differences in interindividual characteristics, base hydration status, etc. To overcome these limitations, semi-quantitative analyses termed “bioelectrical impedance vector analysis” (BIVA) can be conducted based on the measurement of bioelectrical parameters and comparison with reference tolerance or confidence ellipses for reference populations [30]. R and Xc are corrected by height (often termed “classic BIVA”) or cross-sectional areas of the body (“specific BIVA”) [18], plotted in a RXc graph, and compared with 95% confidence limits for mean impedance vectors or percentiles (i.e., 50th, 75th, or 95th) of bioelectrical values of the reference population. Last, localized BIA (L-BIA) has been proposed as a method complementary to MRI to provide field assessment, grading, and monitoring of sport-related injuries through evaluating changes in series-equivalent raw bioelectrical variables (R, Xc, and PhA) in selected muscles compared to non-injured limb measurements following the same principles explained above. A summary of BIA modalities is presented in Figure 2.

## 2. Materials and Methods

To achieve the objectives proposed, a scoping review following the Preferred Reporting Items for Systematic Reviews and Meta-Analyses Scoping Review extension (PRISMA-ScR) [31] was conducted (checklist presented in Supplementary S1). The PROSPERO database is not suitable for scoping reviews, and no formal protocol was registered online for the present work. A preliminary search was conducted in Google Scholar on 26 March 2025 (Supplementary S2) to confirm that no scoping review was already available on the topic, as well as to identify relevant keywords to inform the full search strategy.

A comprehensive search was conducted on 28 March 2025 in four scientific databases (PubMed/MEDLINE, EMBASE, Web of Science (WoS), SPORT Discuss), using search strings consisting of combinations of the relevant key terms, Boolean operators, and wildcards presented in Table S1 (Supplementary S2). Only English-written records published from inception to 28 March 2025 were considered for inclusion. Gray literature and unpublished theses were not specifically searched.

Citations were retrieved and managed in the software EndNote X7^®^. Duplicates were initially removed using the software’s automatic tool, followed by manual removal for the remaining duplicated records. Identified records were independently screened by two experienced reviewers, and any potential disagreement was resolved by consensus and discussion with a third reviewer when needed. Evidence sources included cross-sectional, case-report, and retrospective or prospective observational studies. Excluded sources consisted of intervention studies (beyond routine football training and competition), reviews, editorial letters, study protocols, and congress/conference abstracts or other communications. For full-text record selection, the following population, concept, and context (PCC) framework was followed to guide the inclusion criteria:

- Population: Healthy or injured (sport-related) professional or semi-professional football players ≥ 16 years old (mean), independent of playing position, sex, or ethnicity.
- Concept: Application of bioelectrical impedance analysis (BIA) to estimate body composition outcomes or report/analyze raw bioelectrical parameters.
- Context: Routine training and match-play context in professional and semi-professional football, independent of the competitive or non-competitive period (i.e., off-season, pre-season, in-season).

Studies conducted in different contexts (e.g., exceptional detraining periods during the COVID-19 pandemic) and in different populations (e.g., players <16 years old) were not considered for inclusion. Additional exclusion criteria consisted of (1) studies not reporting league category (e.g., English Premier League) or playing level (e.g., elite level, semi-professional level, national level, etc.); (2) studies recruiting only players at college or amateur levels; (3) studies reporting BIA-derived body composition estimates to describe the efficacy of interventions beyond normal football training and competition (e.g., pharmacological interventions); and (4) studies merely reporting BIA-derived quantitative body composition estimates without further comparison with or validation against different techniques. Notwithstanding, insights from excluded articles were considered for narrative discussion when relevant.

Data were charted through an iterative process, during which the following items were agreed to be relevant and were independently extracted by the same two reviewers: authors and citation, year of publication, location where the study was conducted (origin), primary objectives, population of study and sample size at recruitment, BIA-derived outcomes and measurement time (e.g., pre-season, in-season, off-season), device and configuration, and key findings related to BIA. Further data extraction (e.g., values of body composition outcomes or raw bioelectrical parameters) was reported in tables for narrative discussion when appropriate. Quality appraisal is not a mandatory component of scoping reviews and was considered unnecessary for achieving the objectives of this study.

No quantitative data synthesis (meta-analysis) was performed. Instead, a narrative synthesis of the main findings was provided. Tolerance ellipses, based on the mean values of raw bioelectrical parameters (RXc-graph) for professional and semi-professional male football players after pre-season training, were generated using the BIVA software developed by Piccoli and Pastori [32] and were presented as figures.

## 3. Results

### 3.1. Identification, Screening, and Record Selection Process

**Figure 3:**
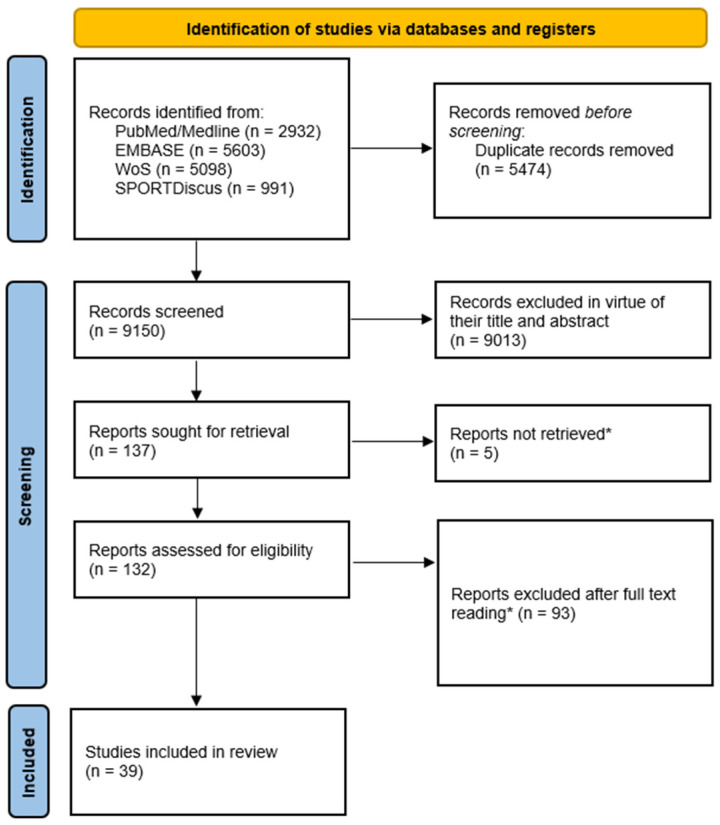
Preferred Reporting Items for Systematic Reviews and Meta-Analyses (PRISMA) flow diagram. * Non-retrieved citations, excluded record, and reasons for exclusion are reported in Supplementary S3.

The PRISMA flow diagram summarizing the processes of record identification, screening, and selection is presented in Figure 3. A total of 14,624 records were identified through the search strategy in the four targeted databases (Supplementary S2). After removing duplicates, 9150 records were available for screening. A total of 9013 records were removed in virtue of their title and abstract, leaving 137 to be sought for full-text retrieval. While 132 reports were available for eligibility assessment, five records could not be located online, or were not provided by the authors upon request. Lastly, 93 reports were excluded after full-text reading, leaving 39 studies to be finally included in the review for qualitative data synthesis.

Non-retrieved citations, reports excluded after full-text reading, and reasons for exclusion are provided in Table S2 (Supplementary S3). Specifically, 31 records were excluded due to only reporting quantitative BIA-estimated body composition outcomes without comparison with or validation against other techniques, 14 studies recruiting different athletes did not provide separate analyses in football players, 13 records consisted of abstracts or congress/conference communications, 10 records were not accessible in English, 9 studies recruited players <16 years old (mean age), 6 studies did not clearly specify the players’ competitive level, another 6 studies did not recruit football players, 2 studies focused on contexts different to normal training and competition, 1 study evaluated the effects of a non-routine intervention, and 1 last study did not report any BIA-derived parameter.

### 3.2. Characteristics of Included Studies

Results from the data charting process are presented in Table S3 (Supplementary S4). Publication dates ranged from 2008 [33] to 2024 [34,35,36]. Geographically, the majority of studies were conducted in either Italy (19 studies [14,22,34,35,37,38,39,40,41,42,43,44,45,46,47,48,49,50,51]) or Spain (10 studies [20,36,52,53,54,55,56,57,58,59]), followed by Portugal (4 studies [60,61,62,63]), Brazil (2 studies [19,64]), and 1 study each in Austria [65], Sweden [33], and Turkey [66]. One study did not specific the country where it was conducted [67]. A total of 2581 professional and semi-professional players were enrolled in the reviewed studies, excluding 727 players from three suspected overlapping studies with the smallest sample size [22,44,54,58]. This comprises 2268 male and only 313 female players (one study did not specify the number of participants [56]). It should be noted that the actual number of unique players may be lower due to potential participant overlap in studies conducted by the same research groups within the same football teams. The age of recruited participants ranged between 16.6 and 30 mean years old [19,58]. Among the included reports, 24 enrolled first division players only [14,20,33,35,37,38,39,41,42,43,45,47,48,49,50,51,52,55,56,57,60,62,63,66], 2 studies recruited second division players [34,40], 1 study included only fourth division players [46], and 3 studies involved players belonging to several professional and semi-professional league divisions [22,36,44]. The remaining reports described recruited participants as professional [19], elite [53,54,58], or national or international level players [59,61,64,65,67]. Reported BIA outcomes were heterogenous and included either raw bioelectrical parameters (i.e., PhA, R, Xc, RXc graph) [20,37,39,43,49,50,55,56,57,67], equation-based body composition outcomes (e.g., TBW, ICW, ECW, FM, FFM, etc.) [33,34,36,51,52,53,54,58,59,61,66], or both [14,19,22,35,38,40,41,42,44,45,46,47,48,60,62,63,64,65]. The most frequently used BIA device was BIA-101^®^ (Akern, Florence, Italy. SF 50 kHz) (22 studies) [14,20,22,34,35,37,38,39,40,41,42,43,44,45,46,47,48,50,55,56,57,67]. All studies used either foot-to-hand configuration (single-frequency or multi-frequency BIA) or localized BIA, and only one study explored the application of BIS [33].

### 3.3. Data Synthesis

Following the iterative data charting process, three general applications were identified. These consist of quantitative body composition assessment (15 studies [14,19,33,34,35,36,38,42,51,52,53,54,58,59,61,65,66]), qualitative or semi-quantitative body composition assessment (15 studies [19,22,35,37,38,40,41,44,45,46,47,48,50,65,67]), and assessment of muscle health and function (14 studies [20,38,39,43,47,49,55,56,57,60,61,62,63,64]).

Specific applications/research areas within quantitative body composition assessment were agreed to include (1) validation of different methods for cross-sectional or longitudinal body composition assessment (e.g., FM, FFM) [33,34,35,36,51,52,53,54,58,59,61,66]; (2) development of new predictive equations/models for estimating body compartments [14,42]; and (3) monitoring hydration status (TBW, ICW, ECW) [65]. Similarly, specific applications/research areas in qualitative or semi-quantitative body composition assessment were proposed to include (1) assessment of raw bioelectrical parameters for identification of sport phenotypes in cross-sectional analyses [19,22,35,37,38,41,42,44,45,46,47,48,50,67]; and (2) evaluation of longitudinal changes in raw bioelectrical parameters (changes in BIVA patterns) for identification of dehydration and muscle adaptations [19,37,38,41,46,47,50,65]. Last, specific applications/research areas in muscle health and function assessment were concluded as follows: (1) comparing ICW/ECW and PhA with indicators of player’s performance or external/internal load (GPS metrics, countermovement jump test (CMJ) scores, biomarkers of exercise-induced muscle damage) [38,39,49,60,62,63,64]; (2) use of localized BIA to detect adaptations in specific muscle groups, or facilitating the monitoring and diagnosis of muscle injury [20,43,45,47,55,56,57].

## 4. Discussion

In the present scoping review, we aimed to map the scientific evidence on the applications and research areas of the bioelectrical impedance technique for monitoring professional and semi-professional football players. A total of 39 records were analyzed and three main general applications and seven specific applications/research areas were identified following the data charting process, which are discussed in the following sections.

### 4.1. Quantitative Body Composition Assessment

As shown in the excluded records (Supplementary S3), studies have often used BIA in isolation to provide estimates of body composition outcomes in football players and/or evaluate the impact of interventions. BIA has been frequently used for body composition assessment purposes (e.g., estimation of FM and FFM) in elite football due to its convenience for quick in situ analyses, particularly during pre-season following off-season detraining [68]. Short-term and long-term detraining are, respectively, defined as fewer than or more than four weeks of partial or complete loss of training-induced adaptations due to training reduction or cessation [69]. Off-season in professional football is a non-competitive period of rest and reduced training load that lasts between 4 and 6 weeks. Long-term detraining such as that seen off-season may be associated with small increases in percentage FM and large decreases in FFM in professional players [70], whereas shorter detraining periods coupled with individualized training prescription might help to attenuate these changes [71]. Increased FM/%FM after off-season inversely correlates with agility and flexibility scores and jump and sprint performance in professional and semi-professional football players [72,73]. Further, a lower fitness level is associated with poor body composition parameters during pre-season, which is frequently stated to contribute to a higher injury rate in-season [74], yet recent analyses have failed to identify associations of FM or FFM at the beginning of pre-season with incidence or injury burden, probably due to the high homogeneity of the players assessed [9,75]. Body composition assessments are particularly useful during pre-season to evaluate detraining-induced changes in FM and FFM after off-season and help design pre-season training programs. In this sense, pre-season training programs (typically 4–6 weeks) are fundamental to rectify increments in FM and decrements in FFM resulting from off-season and cope with in-season fitness requirements [76]. Differences between body composition outcomes at the beginning of pre-season and the end thereof can be used to complement performance measurements and evaluate players’ fitness upon returning to competition.

#### 4.1.1. Cross-Method Validation of BIA Quantitative Assessments

Studies comparing BIA with anthropometric equations and/or DXA, which is considered the gold standard for field monitoring, in professional and semi-professional football players (≥16 years old) are relatively scarce. Based on these studies, it seems clear that cross-sectional BIA analyses notably underestimate %FM compared to DXA values (Table 1), while findings from anthropometric assessments strongly rely on the equations considered [35,36,51,52,53,59,66]. Only research from Munguia-Izquierdo et al. concluded that BIA (MF-BIA: InBody 770^®^, Biospace, Seoul, South Korea & SF-BIA: BC-418^®^, Tanita Corp., Tokyo, Japan) tended to significantly overestimate %FM compared to DXA measurements in elite male young football players (17.1 ± 0.5 years old), albeit only DXA absolute %FM values were reported and notable differences between BIA devices were observed [53].

Aside from FM, BIA is commonly reported to have an error margin of 3–6% when estimating FFM [18]. This aligns with findings from some reviewed studies comparing BIA to DXA, which show that SF-BIA/MF-BIA tends to overestimate FFM (kg) (e.g., 2.5–4.5%) relative to DXA in professional and semi-professional football players (Table 1) [34,58]. This may translate to a lack of accuracy needed for interpreting health and optimizing performance in this population [4]. For instance, some reviewed studies have explored SF-BIA to evaluate body composition changes in response to pre-season training. In first and second division female football players, SF-BIA (BC-420 S MA Master Class III^®^, Tanita Corp., Tokyo, Japan) failed to detect significant FFM or muscle mass gains across playing positions, while significant increases in muscle (kg) and percentage muscle mass were observed using anthropometric equations validated for female athletes [36]. In another recent comprehensive analysis of 21 Serie B (second division) Italian male football players, DXA and different anthropometric equations (e.g., Reilly) were proved successful to detect changes in FFM over the course of a competitive season; however, MF-BIA (InBody 770^®^, Biospace, Seoul, South Korea) did not identify such changes across different time points [34]. As shown in previous studies using DXA, decrements in FM and increases in FFM/LST are expected in elite football players from the beginning of the pre-season to the end thereof [76]. In particular, relatively large decreases in FM as well as moderate increases in FFM and LST are expected from the beginning of the season to mid-season, especially in lower limbs [77]. The utility of BIA to provide accurate repeated estimations throughout these periods seems questionable to date, and whether correlations with different techniques shown in group analyses can be translated to measurements useful for individual players’ assessments is unclear.

#### 4.1.2. Development of New Predictive Equations/Models

While interpreting body composition outcomes (e.g., FFM and FM) estimated by BIA devices may be fast and convenient, it must be considered that BIA devices use equations that often lack transparency [78] and have not been specifically validated for professional football players. Recently, Campa et al. recruited Italian Serie A (first division) male players during the first half of a competitive season and performed DXA and whole body (foot-to-hand) 50 kHz SF-BIA assessments (BIA 101 BIVA PRO^®^, Akern, Florence, Italy). The authors validated BIA-based estimation of FFM, lean soft tissue (LST), and appendicular lean soft tissue (ALST) against DXA values using a train–test validation approach [14]. The resulting predictive models were shown to be more accurate than previous general equations validated for athletes [79]. In a previous research study, Campa et al. developed a model for estimating the three somatotype components in first division football players using SF-BIA and equations validated for athletes (FFM, FM, and %FM) to reduce the number of measurements needed by the classic Heath and Carter method [40]. While novel equations validated for elite football players might help improve the validity of quantitative assessments and somatotype evaluations for that specific population, BIA-derived estimations of body composition outcomes are still subject to different confounding factors that can compromise the accuracy of the individual measurements. Most notably, pre-measurement hydration status can significantly impact BIA results due to changes in TBW and electrolyte content [80]. Further, BIA quantitative measurements depend on assumptions of tissue resistivity, FFM hydration fraction, application of population means to individual assessments, among different factors [81], which help to explain the overall preference for DXA in current guidelines [82]. Last, a recent analysis conducted in 73 athletes observed significant differences in raw bioelectrical parameters measured using three different BIA devices (ImpediMed^®^ SFB7 (BIS) and ImpediMed^®^ SOZO (BIS), Brisbane, Queensland, Australia, and Inbody S10^®^ (MF-BIA), Biospace, Seoul, South Korea), which translated to differences in estimated body composition outcomes, thus adding a further layer of complexity when applying mathematical models and validated predictive equations [83].

#### 4.1.3. Hydration Assessment

An advantage of BIA is the capacity to provide quantitative estimations of TBW using tracer dilution techniques as validation criterion, which may be used to assess changes in body fluid balance through repeated measurements [84]. Among reviewed studies, only one specifically aimed to evaluate changes in TBW throughout a tournament in football players, yet no comparison with other hydration field monitoring methods (e.g., specific urine gravity, body weight loss, etc.) was provided [65]. While TBW measurements performed with BIA and the isotope dilution technique (gold-standard method for laboratory settings) are typically correlated, these correlations are only valid for euhydrated individuals under standardized conditions [80]. Factors such as ICW and ECW equilibration, blood redistribution, sweat, and skin temperature are known to impact TBW measurements after exercise, which, along with non-optimal controlling of measurement conditions (i.e., electrode placement, subject posture, etc.), are proposed to lead to an estimated error range of 3.5–6.9% TBW, which corresponds to a difference of 1.47 to 2.9 L in a 70 kg individual with 60% TBW [80]. Also, BIA has been proposed to be insensitive to acute TBW changes induced by hyperhydration protocols [85]. More recent equations have been validated for estimating TBW and ECW, using dilution techniques as comparison, for athletic populations using 50 kHz SF-BIA (BIA 101^®^, Akern, Florence, Italy), with previous equations showing estimated errors around ±2.5 kg for TBW, ±2 kg for ECW, and ±3 kg for ICW [84]. Alternatively, BIS has been explored to separately assess TBW, ECW, and ICW in hydrated and hypohydrated athletes [86]. While measurements typically correlate well with values obtained using reference methods, their accuracy is compromised when assessed at the individual level [86]. Since 2% dehydration (total body weight loss) is well-accepted as the threshold value for performance impairments in professional football [87], the application of BIA to the monitoring of exercise-induced dehydration in individual players is discouraged due to the relatively large estimated errors and potential lack of correlation with different methods [88].

### 4.2. Qualitative and Semi-Quantitative Body Composition Analysis

In recent years, the analysis of raw bioelectrical variables namely R, Xc, and PhA has gained great attention since these can be directly interpreted without validated regression equations that might compromise the accuracy of the estimations. Furthermore, novel indices derived from these parameters, such as the Levi’s Muscle Index (LMI), have emerged in the literature, demonstrating strong correlations with muscle mass in athletes independent of confounding factors such as body weight, ethnicity, age, and sex [44]. Raw bioelectrical parameters can also be plotted in a RXc graph standardized by height (classic BIVA) or cross-sectional areas (specific BIVA). In cross-sectional classic BIVA, R is lower with higher amounts of electrolyte-containing tissues such as blood and muscle while R tends to augment with greater fat content [89]. Conversely, Xc directly correlates with BCM and, consequently, associates with LST. Regarding repetitive assessments, lengthening in the impedance vector is typically driven by dehydration while shortening would indicate hyperhydration [90]. On the other hand, vector direction (PhA) is impacted by changes in the ICW:ECW ratio, which is indicative of altered membrane integrity [90]. These principles underlie the use of classic BIVA to evaluate sports phenotype and assess changes in water balance in athletes [7]. Alternatively, specific BIVA was introduced in 2012 in an attempt to adjust for body geometry in BIVA assessments [91]. Specific BIVA provides comparable information on ICW:ECW (PhA), but it is proposed to better correlate to changes in FM compared to classic analyses [92]. However, these adjustments make specific BIVA less accurate to evaluate TBW changes [93], highly sample-specific, and its application in sports settings is limited to date [18].

#### 4.2.1. Assessment of Professional/Semi-Professional Football-Specific Phenotypes

**Figure 4:**
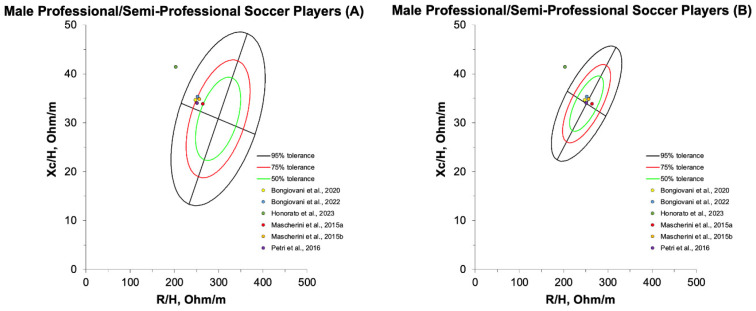
RXc plots of professional and semi-professional football players at end of pre-season compared to healthy adult (**A**) and athlete (**B**) reference tolerance ellipses [19,37,46,47,50,100]. R, resistance; Xc, reactance.

Compared to healthy adults, training-specific adaptations in athletes are reflected in classic BIVA plots by a vector shift to the left in the minor axis, thus indicating higher BCM compared to the general population [94]. Sex- and sport discipline-specific Z, R, and Xc have been reported in studies enrolling individual sport and team sport athletes [95]. Changes in somatotype characterizing players from different team ball sports (i.e., rugby vs. football) are also associated with differences in RXc graph features [42]. Categories within the same sport discipline are also known to impact BIVA ellipses with a shift to the left in the R/H component and a consequent increase in PhA being apparent in elite football compared to lower divisions [22]. This may indicate higher ICW probably due to muscle hypertrophy [22], and is overall aligned with research showing category-based differences in group BIVA mean impedance vectors in different team sports [96]. This evidence supports the validation of specific tolerance ellipses for elite male football players [14,22]. Since body composition is typically highly homogeneous between different playing positions (excluding the goalkeeper) in elite football [97], these ellipses might prove useful when used as “target zones” for BIVA assessments in outfield players [7]. As explained in the following section, changes in tolerance ellipses have been suggested to be period-specific [37], and these may be particularly informative following pre-season training to assess the efficacy of the training microcycle. Data on raw bioelectrical parameters reported in reviewed studies conducted in professional and non-professional football players are presented in Table S4 (Supplementary S5). Tolerance ellipses for professional and semi-professional players obtained at the end of pre-season/at the beginning of the competitive season, plotted against healthy adult [98] and athlete [99] reference ellipses using the BIVA software [32], are represented in Figure 4.

#### 4.2.2. Longitudinal Changes in Bioelectrical Impedance Vector Analysis

Reviewed studies have reported changes in raw bioelectrical parameters across a football tournament [65], throughout different phases of a competitive season [37,38,47,50], across two consecutive menstrual cycles in female players during off-season [41], and in response to pre-season training [19,46,47,50]. Some of these reports observed significant vector changes across pre-season and at different time points during the competitive season, yet findings are often inconsistent. Whole body vector shortening indicative of increased fluid content has been observed in response to pre-season training in a couple of studies [19,46], with authors linking such increments to higher muscle glycogen content [46]. On the other hand, different reports observed no change in any raw bioelectrical parameter throughout this period [47,50]. In the same fashion, some studies have observed a shift to the left in the impedance vector at the beginning of the season compared to the first half of the season, which was argued to be caused by increased muscle mass resulting from pre-season training [37]. However, long-term changes in the impedance vector throughout the season have been notably inconsistent in different studies [38,46,50].

Regarding training-independent changes, BIVA was shown to be effective to detect fluid accumulation resulting from the early follicular phase in female players during off-season [41]. Also, Gatterer et al. reported that BIVA (BIA 2000-M^®^ (MF-BIA), Data Input GmbH, Frankfurt, Germany) was capable of detecting significant fluid loss (vector lengthening) in response to a football tournament in male players [65]. In this sense, reviews on the topic have argued that neither classic or specific BIVA appear to be sensitive enough to identify meaningful dehydration in athletes [18]. However, BIVA may have some advantages over BIA for individual hydration assessment, most notably independence of regression equations and theoretical models [27]. In fact, reductions in Z/height vector changes have been shown to be associated with increased TBW and ICW measured using gold-standard methods in athletes of different sports [37]. Vectors positioned within the 50% tolerance ellipse of population-specific reference plots are generally considered indicative of normal hydration and cellular mass. Regarding hydration classification, a progressive lengthening of the vector from the 51st to the 75th percentile suggests dehydration, while values exceeding the 76th percentile indicate more severe dehydration [27]. Conversely, vector shortening within the same percentile ranges on the lower end of the plot is indicative of hyperhydration, with values exceeding the 76th percentile denoting more pronounced hyperhydration [27].

As mentioned, athletes are typically recommended to avoid fluid deficits >2% body mass [101], yet around 66.2% of professional football players engage in exercise in a hypohydrated state [102], which further aggravates exercise-induced dehydration. Pre-training classic BIVA assessment might be a valuable tool to screening players at risk of hypohydration. However, open questions remain as to whether vector lengthening outside the 50th percentile for the population-specific reference tolerance ellipse can effectively identify hypohydrated professional football players [18]. In particular, research is warranted to determine whether BIVA can sensitively detect moderate fluid deficits (e.g., 2–5%), which are associated with impaired exercise performance. It is also worth noting that measuring exercise-induced dehydration through BIA/BIVA immediately after exercise can be confounded by the effect of exercise on cutaneous blood flow, skin temperature, and electrolyte accumulation [103], and is typically discouraged. Additional confounding factors not only include inter-device variability—particularly differences among 50 kHz phase-sensitive SF-BIA, MF-BIA, and BIS technologies [104]—but also the position, size, and material composition of the electrodes applied, with low intrinsic impedance electrodes being preferable [105,106].

### 4.3. Assessment of Muscle Health and Performance

Raw parameters namely R, Xc, and PhA can also be independently interpreted to assess cell membrane integrity status. Specifically, PhA is the angle formed between R and Xc, and has been proposed as an independent indicator of muscle quality—classically defined as strength relative to muscle mass [107]. In athletes, whole body PhA has been reported to correlate both with relative power and with relative and absolute strength [108]. Similarly, athletes typically display higher PhA than the general population. For instance, a reference PhA of 7.6° (50th percentile) has been reported in male football players (BIA-101^®^ (SF-BIA), Akern, Florence, Italy) [67], while large meta-analyses have shown mean PhA values in healthy male adults (18–48 years old) ranging between 6.9 and 7.2° [109].

#### 4.3.1. Phase Angle as an Indicator of Player Load and Performance

Some authors have explored PhA as an indicator of muscle performance in professional and semi-professional football players. Associations with sprint performance have been reported in cross-sectional analyses including football players participating in national and international events [64]. Bongiovanni et al. reported that in-season regional lower hemisome PhA was more strongly associated with jump (countermovement jump (CMJ)) and sprint performance than upper hemisome or whole body PhA (BIA-101 BIVA PRO^®^ (SF-BIA), Akern, Florence, Italy) [100]. These findings led the authors to suggest that lower body regional BIA is more informative for the monitoring of lower body neuromuscular performance in these participants [100]. In the same vein, an increase in lower body PhA from the beginning of pre-season to the second half of the competitive season was shown to be associated with CMJ performance in first division Italian male football players (BIA-101 BIVA PRO^®^ (SF-BIA), Akern, Florence, Italy) in a different trial from the same group [39]. Associations between CMJ, VO_2_max, and PhA have been recently reported in female professional players [62]. Based on these studies, PhA appears to be a promising parameter to supplement muscle performance measurements, particularly after training programs; however, evidence on adult professional or semi-professional football players remains limited. For instance, Custodio-Martins et al. observed that whole body PhA (InBody 720^®^ (MF-BIA), Biospace, Seoul, South Korea) was a predictor of 10 m and 30 m sprint times and repeated sprint ability after adjusting for age and body composition (body fat and LST) in ≈15-year old male football players at the end of pre-season [110]. Hetherington et al. observed that whole body PhA and regional PhA (BIA-101^®^ (SF-BIA), Akern, Florence, Italy) were positively associated with indicators of absolute and relative power and strength after adjusting for age, sex, height, and sport discipline in a cohort of adult athletes, including 12 football players, yet no differences between PhA types were shown and no separate analysis was performed in football players [108]. Further research in elite adult players is needed to better understand PhA as a potential index of muscle performance in this population.

The role of PhA as a marker of cellular integrity has led some authors to suggest that changes in PhA driven by altered reactance at low frequencies might potentially indicate substantial exercise-induced muscle damage (EIMD) [111], which might help support different marker assessments. Evaluating objective markers of EIMD is a topic of great scientific interest in professional football in order to support functional and perceptual tests and guide recovery protocols [112]. To date, research in this field has been limited to biochemical molecules that might not completely discriminate localized damage and rely on specific clearance kinetics with high inter-individual variability [112]. Only Moya-Amaya et al. have evaluated associations between PhA and EIMD biomarkers during post-exercise recovery in professional football players [49]. Unexpectedly, the authors observed direct correlations between PhA and different EIMD biomarkers (creatine kinase (CK), lactate dehydrogenase (LDH), c-reactive protein (CRP)) at 36 h post-match. However, no significant change in PhA was observed before and after the matches, measurements were only conducted before and 36 h into post-match recovery, and only whole body PhA at 50 kHz was reported (MC-780 MA^®^ (MF-BIA); Tanita Corp, Tokyo, Japan) [49]. These findings are not aligned with other studies conducted in different populations. In particular, Yamaguchi et al. recruited 35 healthy male non-trained adults who underwent a single-arm eccentric exercise routine [111]. EIMD markers, including maximum voluntary contraction (MVC), visual analog scale (VAS) pain assessment, range of motion (ROM), and urinary titin n-terminal fragment excretion (UTF), were assessed before and after exercise (0 h, 1 h), and up 168 h into the recovery period [111]. The authors also measured segmental bioelectrical impedance parameters (regional and whole body R, Xc, PhA, and estimated ICW, ECW, and TBW (InBody 720^®^ (MF-BIA), Biospace, Seoul, South Korea)) throughout the follow-up period [111]. Interestingly, temporal changes in regional PhA and Xc indirectly correlated with UTF only in the exercised arm [111]. An increase in ECW and TBW without a decrease in ICW was apparent immediately after and between 24 h and 168 h into recovery [111]. Altogether, the authors suggested that BIA changes in regional Xc were driven by sarcolemmal disruption due to the onset of EIMD, while changes in ECW/TBW were related to acute and long-term inflammatory processes [111]. While these are interesting findings, whether changes in BIA parameters are sensitive enough to discriminate post-match EIMD in football players is still unknown.

Last, the reviewed studies exploring associations between PhA and different internal load (e.g., RPE scores) and external load metrics (e.g., GPS variables) have reported mixed results. In particular, an inverse association between fatigue and PhA was reported by Nabuco et al. in male players [64], whereas no significant relation was shown in female players in the study conducted by Olivera et al. [63]. Similarly, Fernandes et al. reported no association between any evaluated GPS variable and PhA or ICW/ECW [60], while Oliveria et al. observed associations between high speed running, ECW/ICW, and PhA [62]. All these studies used (MF-BIA) Inbody S10^®^ (Biospace, Seoul, South Korea). Future studies evaluating time-course analyses of post-match recovery markers might benefit from including BIA assessments and external load metrics to elucidate whether bioelectrical parameter analysis might help supplement the information provided by recovery measurements.

#### 4.3.2. Localized Bioelectrical Impedance Analysis

A promising application of bioelectrical impedance in professional football is the monitoring and classification of soft tissue injuries through series-equivalent L-BIA. Early experiments conducted by Henry Lukaski established the foundation for using localized analysis of raw bioelectrical impedance parameters to evaluate the effects of structural component disruption on the electrical properties of biological tissues [28]. Complete disruption of cellular membranes leads to Xc values close to zero as well as increments in R resulting from higher ECW component, while localized fluid accumulation decreases R and Xc without proportionally impacting PhA [113]. Findings from a pilot study on professional football players indicated that decreases in R, Xc, and PhA, measured using L-BIA at three different times—30 min before training, 24 h after injury, and during recovery—were associated with injury severity as graded by MRI [113]. These results are consistent with the hypothesis that post-injury R decrements are associated with blood accumulation at the injury site, while Xc and PhA values decrease due to muscle cell damage [113]. In an ensuing study, 21 muscle injuries (hamstring, quadricep, and adductor) were studied in professional football players through L-BIA (24 h after injury) and MRI, and percentage differences in raw bioelectrical parameters were compared to the non-injured leg [56]. A significant decrease in Xc was observed with all injury grades (I–III), while decreases in PhA and R were dependent on the grade of the injury [56]. In another analysis from the same group, 37 muscle injuries were evaluated by MRI and L-BIA at 24 h after the injury, including 4 tendinous, 7 myofascial junction, and 26 myotendinous junction injuries [55]. Clear differences in R, Xc, and PhA between injured and contralateral non-injured sides were apparent only in myofascial and myotendinous junction injuries [55]. Changes in bioelectrical parameters are also associated with injury grade (I–III) in myotendinous injuries, with decrements in Xc the parameter being the best for discriminating injury grades [55]. According to these authors, muscle fiber retraction denoted by changes in Xc might be better correlated with injury grade compared to the presence of edema assessed through MRI-based criteria [57].

Overall, the studies discussed above support Xc as the most sensitive parameter, consistently decreasing across all injury grades and discriminating between severity levels. PhA, reflecting cell membrane integrity, may also decline post-injury, particularly in more severe cases. Also, R tended to decrease (to a lesser extent than Xc), therefore indicating localized fluid accumulation. Together, these parameters provide a non-invasive means to support injury diagnosis and monitor healing. Since myotendinous injury grade has been shown to directly associate with return to play time [55], L-BIA might be a valuable tool to help evaluate return to competition. However, L-BIA is not exempt from limitations namely the lack of standardization of BIA devices and low intrinsic impedance surface electrodes, challenges inherent to the identification of different injury types (e.g., anatomically deep injuries), and the impact of heterogenous electrode placement in follow-up measurements [113].

Studies reporting differences between injured and non-injured limbs/baseline values 24 h after injury diagnosis in professional football players are presented in Table 2. All these studies used BIA-101^®^ (SF-BIA), Akern, Florence, Italy.

Last, L-BIA might also offer opportunities for the assessment of muscle group-specific training adaptations. Significant changes in hamstring, quadricep, and calf L-BIA parameters, particularly R/length, were reported after a 50-day pre-season training (BIA-101^®^ (SF-BIA), Akern, Florence, Italy) [47]. Additionally, in a recent trial, Honorato et al. observed an increase in hamstring-specific local PhA from the beginning to the end of pre-season (six weeks) in professional football players, while whole body PhA was not significantly impacted by the training program (Quantum V Segmental BIA^®^ (SF-BIA), RJL Systems, Clinton Township, MI, USA) [19]. These results are promising, but further studies are warranted to evaluate the implications of localized hamstring PhA assessment in the evaluation of pre-season training adaptations and in the prevention of potential injuries in specific muscle groups.

The present scoping review is not exempt from limitations. Particularly, no specific search of gray literature was conducted, and language was limited to English, which might have led to the exclusion of relevant sources of information. As is frequent in scoping reviews, no quality appraisal or meta-analysis were conducted, which may limit the ability to weight the strength of the evidence. Limitations of the underlying evidence also include heterogeneity of included studies, most notably the overrepresentation of male athletes, limited validation with reference techniques in body composition assessments, and potential participant overlap across reviewed studies. Also, analyzed studies used a diversity of bioelectrical impedance devices, each employing proprietary algorithms, which introduces variability and potential error in the reported outcomes. A key limitation in the evaluation of changes through longitudinal BIA assessments—quantitative body composition, hydration/rehydration, and muscle health and adaptations—is the intra- and inter-individual variability of the measurements, which must be lower than the magnitude of the observed changes to ensure meaningful interpretation [114,115]. As an example, day-to-day measurement errors of up to 1% FM have been reported for some devices [116], potentially limiting their sensitivity to detect small but physiologically relevant changes. Best practices to improve the usefulness of these measurements may include following standardized protocols, using multi-day average values rather than single estimates, and using the same device in repeated assessments [115]. On a practical note, subject-related factors (e.g., hydration status) as well as environmental conditions, including temperature, may interfere with BIA measurement [117]. These variables pose a challenge to obtaining consistent and reliable assessments when implementing BIA in routine monitoring protocols.

## 5. Conclusions

Bioelectrical impedance analysis (BIA) has gained great attention in recent years as a double-indirect, non-invasive, fast, and convenient tool for the quantitative and semi-quantitative repeated assessment of body composition outcomes in professional football players. This scoping review highlights the evolving role of BIA in professional and semi-professional football, mapping its applications across quantitative body composition assessment, semi-quantitative evaluation of hydration and cellular mass and integrity, and monitoring of muscle health and injury. Evidence suggests that field quantitative estimations of fat mass, fat-free mass, and lean soft tissue as well as hydration-related outcomes might lack sufficient accuracy needed for decision-making processes in professional and semi-professional football settings. Novel equations specifically validated for elite football players might help improve the accuracy of these predictions, yet different limitations inherent to the technology may still compromise its widespread application. Conversely, recent applications of BIA involving the analysis of raw bioelectrical parameters to provide semi-quantitative interpretations of hydration, body cell mass, and membrane integrity outcomes might prove useful to evaluate potential target zones for body composition in high level football players and supplement different assessments aimed to guide hydration and recovery protocols, support the diagnosis and prognosis of football-related injuries, and facilitate return to competition. However, these potential applications would benefit from further research—also including female players—evaluating normal ranges for hydration classification in bioelectrical impedance vector analyses, correlations between raw parameters and post-match recovery markers, associations between segmental and whole body parameters with muscle performance metrics, and standardization of measurement protocols in localized BIA assessments.

## Figures and Tables

**Table 1:** Studies reporting %body fat mass and fat-free mass (kg) assessed by DXA or estimated by BIA in professional and semi-professional football players.

Reference	Characteristics of Participants and Measurement Time	BIA Device and Configuration	Fat Mass (%)	Fat-Free Mass (kg)	Agreement Between Calculated (DXA) and Estimated (BIA/BIS) Outcomes
Svantesson et al., 2008 [33]	Swedish first division male soccer players (*n* = 17; 24.1 ± 3.8 years old) (mean ± SD). (Timing not specified.)	Hydra 4200^®^, Xitron Technologies Inc., San Diego, CA, USA. Foot-to-hand. BIS.	DXA: 10.9 ± 3.5 BIS: 9.7 ± 3.6	DXA: 72.4 ± 6.2 BIS: 72.8 ± 7.9	BIS underestimates mean %FM by ~1.1% in professional players compared to DXA, albeit differences were not statistically significant. Agreement at individual level of BIS estimations was highly variable.
Leao et al., 2017 [61]	U19 National level male football players (*n* = 25; 17.28 ± 0.54 years old) (mean ± SD). In-season.	BC-418^®^, Tanita Corp., Tokyo, Japan. Foot-to-hand. SF-BIA.	DXA: 14.16 ± 1.91 SF-BIA: 11.97 ± 2.66	--	Only moderate correlations were found between DXA-calculated and SF-BIA-estimated %FM, with the latter technique underestimating %FM by 2.21% in U19 players (statistical significance not reported).
Suarez-Arrones et al., 2019 [71]	Italian first division male soccer players (*n* = 18; 27.6 ± 3.0 years old) (mean ± SD). End of the competitive season.	MC-180 MA III^®^, Tanita Corp., Tokyo, Japan. Foot-to-hand. MF-BIA.	DXA: 14.4 ± 1.3 MF-BIA: 9.5 ± 2.6	--	An unclear correlation was concluded between MF-BIA-estimated and DXA-calculated %FM values in professional players. MF-BIA substantially underestimated %FM compared to DXA. Differences were qualitatively classified as almost certainly lower than DXA.
Núñez et al., 2020 [58]	Spanish elite male football players (*n* = 40; 16.6 ± 0.5 years old) (mean ± SD). Pre-season (T0) and mid-season (T1).	Inbody 770^®^, Biospace, Seoul, South Korea. Foot-to-hand. MF-BIA. and BC-418^®^, Tanita Corp., Tokyo, Japan. Foot-to-hand. SF-BIA.	--	DXA: 55.73 ± 4.04 (T0), 56.79 ± 4.15 (T1) MF-BIA (Inbody): 57.09 ± 4.61 (T0), 58.98 ± 4.77 (T1) SF-BIA (BC-418): 56.31 ± 4.24 (T0), 57.09 ± 4.38 (T1)	Tanita SF-BIA showed no statistically significant standardized difference compared to DXA-derived FFM values, whereas InBody MF-BIA demonstrated a significant standardized difference, indicating lower agreement with DXA. Both BIA techniques showed positive and very large correlations with DXA and identified significant changes in FFM from pre-season to mid-season in professional players.
Martinez-Ferran et al., 2022 [52]	Spanish first division male football players (*n* = 21; 26.3 ± 3.7 years old) (mean ± SD). First half of the competitive season.	BC-545N^®^, Tanita Corp, Tokyo, Japan. Foot-to-hand. SF-BIA.	DXA: 15.3 ± 2.0 MF-BIA: 13.0 ± 2.5	--	MF-BIA %FM only showed a moderate correlation with DXA, and standardized differences were qualitatively classified as almost certainly lower than DXA, indicating a consistent underestimation of %FM in professional players.
Tornero-Aguilera et al., 2022 [59]	National-level male (*n* = 70; 21.8 ± 5.0 years old) and female football players (*n* = 76; 22.2 ± 3.2 years old) (mean ± SD). Before pre-season.	Inbody 770^®^, Biospace, Seoul, South Korea. Foot-to-hand. MF-BIA.	DXA: 19.0 ± 3.7 (male), 29.2 ± 4.8 (female) MF-BIA: 9.3 ± 4.3 (male), 14.9 ± 5.6 (female)	--	Statistically significant differences were observed between %FM values estimated via MF-BIA and assessed by DXA, with MF-BIA notably underestimating FM in both national-level male and female players.
Bongiovanni et al., 2024 [34]	Italian second division male football players (*n* = 21; 23.7 ± 4.8 years old) (Statistic type unspecified). Four time points throughout the competitive season (October (T0), December (T1), February (T2), April (T3)).	Inbody 770^®^, Biospace, Seoul, South Korea. Foot-to-hand. MF-BIA.	DXA: 12.2 ± 2.2 (T0), 12.4 ± 2.1 (T1), 12.3 ± 2.1 (T2), 12.6 ± 2.3 (T3) MF-BIA (Inbody): 8.4 ±2.8 (T0), 8.6 ± 2.7 (T1), 8.8 ± 2.9 (T2), 8.8 ± 2.7 (T3)	DXA: 70.8 ± 5.1 (T0), 71.6 ± 5.7 (T1), 72.5 ± 6.0 (T2), 71.7 ± 5.4 (T3) MF-BIA (Inbody): 73.9 ± 5.6 (T0), 74.2 ± 5.7 (T1), 74.9 ± 6.4 (T2), 74.3 ± 6.1 (T3)	Despite showing a strong correlation with DXA, MF-BIA demonstrated limited sensitivity in detecting seasonal changes in FFM. In contrast, MF-BIA-derived %FM values were significantly lower than those obtained via DXA, with statistically significant differences and variable agreement reflected in lower correlation coefficients.

Values presented as mean ± standard deviation (SD). BIA, bioelectrical impedance analysis; BIS, bioelectrical impedance spectroscopy; DXA, dual-energy X-ray absorptiometry; MF, multi-frequency; SF, single-frequency.

**Table 2:** Localized bioelectrical impedance analysis data of injured and non-injured limb/baseline values in professional football players.

Reference	Characteristics of Participants and Number of Injuries	Localized Bioelectric Impedance Analysis (L-BIA)				BIA Device and Configuration
Nescolarde et al., 2013 [20]	Spanish first division male football players (*n* = 3 lower limb muscle injuries)	Grade I Muscle Injury (*n* = 1)	Baseline values	Injured (24 h post-injury)	Difference (%)	BIA-101^®^, Akern, Florence, Italy. Muscle Localized. SF 50 kHz.
		R (Ohm)	42	37	−11.9	
		Xc (Ohm)	17	13	−23.5	
		PhA (°)	22	19.3	−12.3	
		Grade II Muscle Injury (*n* = 1)	Baseline values	Injured (24 h post-injury)	Difference (%)	
		R (Ohm)	68	54	−20.6	
		Xc (Ohm)	19	13	−31.6	
		PhA (°)	15.6	13.5	−13.5	
		Grade III Muscle Injury (*n* = 1)	Baseline values	Injured (24 h post-injury)	Difference (%)	
		R (Ohm)	67	51.5	−23.13	
		Xc (Ohm)	20	11	−45	
		PhA (°)	16.6	12	−27.7	
Nescolarde et al., 2014 [56]	Spanish first division male football players (*n* = 21 lower limb muscle injuries)	Grade I Muscle Injury (*n* = 11)	Non-injured limb	Injured (24 h post-injury)	Difference (%)	BIA-101^®^, Akern, Florence, Italy. Muscle Localized. SF 50 kHz.
		R (Ohm)	40.4 ± 9.2	36.1 ± 7.6	−10.4	
		Xc (Ohm)	15.3 ± 1.6	12.7 ± 1.6	−17.5	
		PhA (°)	21.4 ± 3.9	19.9 ± 4	−9.0	
		Grade II Muscle Injury (*n* = 8)	Non-injured limb	Injured (24 h post-injury)	Difference (%)	
		R (Ohm)	37.9 ± 5.9	30.9 ± 4.9	−18.4	
		Xc (Ohm)	15.3 ± 2.5	10.2 ± 1.7	−32.9	
		PhA (°)	22.1 ± 3.5	18.3 ± 2.3	−16.6	
		Grade III Muscle Injury (*n* = 2)	Non-injured limb	Injured (24 h post-injury)	Difference (%)	
		R (Ohm)	44.8 ± 2.7	38.4 ± 1.8	−14.1 ± 9.3	
		Xc (Ohm)	18.3 ± 1.7	8.6 ± 0.1	−52.9 ± 3.6	
		PhA (°)	22.2 ± 0.7	12.7 ± 0.8	−43.1 ± 1.8	
Francavilla et al., 2015 [43]	Italian first division male soccer player (*n* = 1 lower limb muscle injury)	Grade II Muscle Injury (*n* = 1)	Baseline values	Injured (24 h post-injury)	Difference (%)	BIA-101^®^, Akern, Florence, Italy. Muscle Localized. SF 50 kHz.
		R (Ohm)	23.3	21.2	−9	
		Xc (Ohm)	1.7	1.2	−29.4	
		PhA (°)	4.3	3.3	−23.3	
Nescolarde et al., 2017 [57]	Spanish first division male football players (*n* = 22 lower limb muscle injuries)	Grade I Muscle Injury (*n* = 7)	Non-injured limb	Injured (24 h post-injury)	Difference (%)	BIA-101^®^, Akern, Florence, Italy. Muscle Localized. SF 50 kHz
		R (Ohm)	37.3 ± 7.8	33.4 ± 6.6	−10.2	
		Xc (Ohm)	14.9 ± 1.9	12.9 ± 1.7	−13.4	
		PhA (°)	22.2 ± 3.8	21.5 ± 3.6	−3.2	
		Grade II (no gap) Muscle Injury (*n* = 8)	Non-injured limb	Injured (24 h post-injury)	Difference (%)	
		R (Ohm)	42.1 ± 7.8	36.7 ± 7.0	−12.8	
		Xc (Ohm)	15.1 ± 1.8	11.6 ± 1.4	−23.5	
		PhA (°)	20.1 ± 2.9	17.8 ± 2.4	−11.2	
		Grade II (gap) Muscle Injury (*n* = 7)	Non-injured limb	Injured (24 h post-injury)	Difference (%)	
		R (Ohm)	41.2 ± 13.3	32.8 ± 10.3	−19.9	
		Xc (Ohm)	16.2 ± 2.8	10.1 ± 1.9	−37.5	
		PhA (°)	22.4 ± 4.6	17.6 ± 3.1	−20.5	
Nescolarde et al., 2020 [55]	Spanish first division male football players (*n* = 26 lower limb myotendinous junction injuries)	Grade I Muscle Injury (*n* = 11)	Non-injured limb	Injured (24 h post-injury)	Difference (%)	BIA-101^®^, Akern, Florence, Italy. Muscle Localized. SF 50 kHz.
		R (Ohm)	40.4 ± 8.3	37.3 ± 8.4	−7.9	
		Xc (Ohm)	15.1 ± 2.3	13.2 ± 2.3	−12.3	
		PhA (°)	21.0 ± 4.5	20.2 ± 4.6	−4.3	
		Grade II Muscle Injury (*n* = 8)	Non-injured limb	Injured (24 h post-injury)	Difference (%)	
		R (Ohm)	40.5 ± 9.0	36.7 ± 7.0	−8.8	
		Xc (Ohm)	14.6 ± 1.7	11.7 ± 1.1	−19.5	
		PhA (°)	20.3 ± 3.2	18.1 ± 2.9	−10.8	
		Grade III Muscle Injury (*n* = 7)	Non-injured limb	Injured (24 h post-injury)	Difference (%)	
		R (Ohm)	39.2 ± 6.0	32.5 ± 6.0	−17.1	
		Xc (Ohm)	15.3 ± 2.7	10.2 ± 1.7	−32.7	
		PhA (°)	21.6 ± 4.0	17.7 ± 3.1	−17.8	

Values presented as mean ± standard deviation (SD). BIA, bioelectrical impedance analysis; L-BIA, localized bioelectrical impedance analysis; PhA, phase angle; R, resistance; SF, single frequency; Xc, reactance.

## Data Availability

No new data were created or analyzed in this study.

## Supplementary Materials

The following supporting information can be downloaded at: https://www.mdpi.com/article/10.3390/sports13100348/s1. Supplementary S1: PRISMA Checklist; Supplementary S2: Table S1: Full search strategy; Supplementary S3: Table S2: Non-retrieved records and records excluded after full-text reading; Supplementary S4: Table S3: Data charting of included studies; Supplementary S5: Table S4: Data extraction of studies reporting raw bioelectrical parameters.

## References

[1] Ward L.C. (2018). Human body composition: Yesterday, today, and tomorrow. Eur. J. Clin. Nutr..

[2] de la Cruz Marcos S., Redondo del Río M.P., de Mateo Silleras B. (2021). Applications of Bioelectrical Impedance Vector Analysis (BIVA) in the Study of Body Composition in Athletes. Appl. Sci..

[3] Shepherd J.A., Ng B.K., Sommer M.J., Heymsfield S.B. (2017). Body composition by DXA. Bone.

[4] Ackland T.R., Lohman T.G., Sundgot-Borgen J., Maughan R.J., Meyer N.L., Stewart A.D., Müller W. (2012). Current status of body composition assessment in sport: Review and position statement on behalf of the ad hoc research working group on body composition health and performance, under the auspices of the IOC Medical Commission. Sports Med..

[5] Campa F., Gobbo L.A., Stagi S., Cyrino L.T., Toselli S., Marini E., Coratella G. (2022). Bioelectrical impedance analysis versus reference methods in the assessment of body composition in athletes. Eur. J. Appl. Physiol..

[6] Campa F., Toselli S., Mazzilli M., Gobbo L.A., Coratella G. (2021). Assessment of Body Composition in Athletes: A Narrative Review of Available Methods with Special Reference to Quantitative and Qualitative Bioimpedance Analysis. Nutrients.

[7] Lukaski H., Raymond-Pope C.J. (2021). New Frontiers of Body Composition in Sport. Int. J. Sports Med..

[8] Requena B., García I., Suárez-Arrones L., Sáez de Villarreal E., Naranjo Orellana J., Santalla A. (2017). Off-Season Effects on Functional Performance, Body Composition, and Blood Parameters in Top-Level Professional Soccer Players. J. Strength Cond. Res..

[9] Seow D., Massey A. (2022). Correlation between preseason body composition and sports injury in an English Premier League professional football team. BMJ Open Sport Exerc. Med..

[10] Figueiredo D.H., Dourado A.C., Stanganelli L.C.R., Gonçalves H.R. (2021). Evaluation of body composition and its relationship with physical fitness in professional soccer players at the beginning of pre-season. Retos Nuevas Tend. En Educ. Física Deporte Y Recreación.

[11] Bradley P.S., Archer D.T., Hogg B., Schuth G., Bush M., Carling C., Barnes C. (2016). Tier-specific evolution of match performance characteristics in the English Premier League: It’s getting tougher at the top. J. Sports Sci..

[12] Nédélec M., McCall A., Carling C., Legall F., Berthoin S., Dupont G. (2012). Recovery in soccer: Part I—Post-match fatigue and time course of recovery. Sports Med..

[13] Mills C.D., De Ste Croix M.B., Cooper S.-M. (2017). The importance of measuring body composition in professional football players: A commentary. Sport Exerc. Med. Open J..

[14] Campa F., Bongiovanni T., Rossi A., Cerullo G., Casolo A., Martera G., Trecroci A., Moro T., Paoli A. (2023). Athletic bioimpedance-based equations underestimate fat free mass components in male elite soccer players: Development and validation of new soccer-specific predictive models. J. Transl. Med..

[15] Sardinha L.B., Correia I.R., Magalhães J.P., Júdice P.B., Silva A.M., Hetherington-Rauth M. (2020). Development and validation of BIA prediction equations of upper and lower limb lean soft tissue in athletes. Eur. J. Clin. Nutr..

[16] Comfort P., Stewart A., Bloom L., Clarkson B. (2014). Relationships Between Strength, Sprint, and Jump Performance in Well-Trained Youth Soccer Players. J. Strength Cond. Res..

[17] Marini E., Campa F., Buffa R., Stagi S., Matias C.N., Toselli S., Sardinha L.B., Silva A.M. (2020). Phase angle and bioelectrical impedance vector analysis in the evaluation of body composition in athletes. Clin. Nutr..

[18] Castizo-Olier J., Irurtia A., Jemni M., Carrasco-Marginet M., Fernández-García R., Rodríguez F.A. (2018). Bioelectrical impedance vector analysis (BIVA) in sport and exercise: Systematic review and future perspectives. PLoS ONE.

[19] Honorato R.d.C., Soares Marreiros Ferraz A., Kassiano W., Martins P.C., Silva D.A.S., Ceccatto V.M. (2023). Regional phase angle, not whole-body, is augmented in response to pre-season in professional soccer players. Res. Sports Med..

[20] Nescolarde L., Yanguas J., Lukaski H., Alomar X., Rosell-Ferrer J., Rodas G. (2013). Localized bioimpedance to assess muscle injury. Physiol. Meas..

[21] Moon J.R. (2013). Body composition in athletes and sports nutrition: An examination of the bioimpedance analysis technique. Eur. J. Clin. Nutr..

[22] Levi Micheli M., Pagani L., Marella M., Gulisano M., Piccoli A., Angelini F., Burtscher M., Gatterer H. (2014). Bioimpedance and Impedance Vector Patterns as Predictors of League Level in Male Soccer Players. Int. J. Sports Physiol. Perform..

[23] Foster K.R., Lukaski H.C. (1996). Whole-body impedance--what does it measure?. Am. J. Clin. Nutr..

[24] Hoffer E.C., Meador C.K., Simpson D.C. (1969). Correlation of whole-body impedance with total body water volume. J. Appl. Physiol..

[25] Sagayama H., Yamada Y., Ichikawa M., Kondo E., Yasukata J., Tanabe Y., Higaki Y., Takahashi H. (2020). Evaluation of fat-free mass hydration in athletes and non-athletes. Eur. J. Appl. Physiol..

[26] Buchholz A.C., Bartok C., Schoeller D.A. (2004). The validity of bioelectrical impedance models in clinical populations. Nutr. Clin. Pract. Off. Publ. Am. Soc. Parenter. Enter. Nutr..

[27] Lukaski H.C., Vega Diaz N., Talluri A., Nescolarde L. (2019). Classification of hydration in clinical conditions: Indirect and direct approaches using bioimpedance. Nutrients.

[28] Lukaski H.C. (1996). Biological indexes considered in the derivation of the bioelectrical impedance analysis. Am. J. Clin. Nutr..

[29] Buendia R., Seoane F., Lindecrantz K., Bosaeus I., Gil-Pita R., Johannsson G., Ellegård L., Ward L. (2015). Estimation of body fluids with bioimpedance spectroscopy: State of the art methods and proposal of novel methods. Physiol. Meas..

[30] Piccoli A., Rossi B., Pillon L., Bucciante G. (1994). A new method for monitoring body fluid variation by bioimpedance analysis: The RXc graph. Kidney Int..

[31] Tricco A.C., Lillie E., Zarin W., O’Brien K.K., Colquhoun H., Levac D., Moher D., Peters M.D., Horsley T., Weeks L. (2018). PRISMA extension for scoping reviews (PRISMA-ScR): Checklist and explanation. Ann. Intern. Med..

[32] Piccoli A., Pastori G. (2002). BIVA Software.

[33] Svantesson U., Zander M., Klingberg S., Slinde F. (2008). Body composition in male elite athletes, comparison of bioelectrical impedance spectroscopy with dual energy X-ray absorptiometry. J. Negat. Results Biomed..

[34] Bongiovanni T., Lacome M., Rodriguez C., Tinsley G.M. (2024). Tracking Body Composition Over a Competitive Season in Elite Soccer Players Using Laboratory- and Field-Based Assessment Methods. J. Strength Cond. Res..

[35] Petri C., Pengue L., Bartolini A., Pistolesi D., Arrones L.S. (2024). Body Composition Changes in Male and Female Elite Soccer Players: Effects of a Nutritional Program Led by a Sport Nutritionist. Nutrients.

[36] Ramírez-Munera M., Arcusa R., López-Román F.J., Victoria-Montesinos D., García-Muñoz A.M., Ávila-Gandía V., Pérez-Piñero S., Marhuenda J. (2024). Anthropometric and Body Composition Changes during Pre-Season of Spanish Professional Female Soccer Players According to Playing Position. Nutrients.

[37] Bongiovanni T., Mascherini G., Genovesi F., Pasta G., Iaia F.M., Trecroci A., Ventimiglia M., Alberti G., Campa F. (2020). Bioimpedance Vector References Need to Be Period-Specific for Assessing Body Composition and Cellular Health in Elite Soccer Players: A Brief Report. J. Funct. Morphol. Kinesiol..

[38] Bongiovanni T., Tinsley G., Martera G., Orlandi C., Genovesi F., Puleo G., Rossi A., Trecroci A. (2022). Regional Lean Soft Tissue and Intracellular Water Are Associated with Changes in Lower-Body Neuromuscular Performance: A Pilot Study in Elite Soccer Players. Eur. J. Investig. Health Psychol. Educ..

[39] Bongiovanni T., Trecroci A., Rossi A., Iaia F.M., Pasta G., Campa F. (2021). Association between change in regional phase angle and jump performance: A pilot study in serie a soccer players. Eur. J. Investig. Health Psychol. Educ..

[40] Campa F., Bongiovanni T., Matias C.N., Genovesi F., Trecroci A., Rossi A., Iaia F.M., Alberti G., Pasta G., Toselli S. (2020). A New Strategy to Integrate Heath-Carter Somatotype Assessment with Bioelectrical Impedance Analysis in Elite Soccer Player. Sports.

[41] Campa F., Micheli M.L., Pompignoli M., Cannataro R., Gulisano M., Toselli S., Greco G., Coratella G. (2022). The Influence of Menstrual Cycle on Bioimpedance Vector Patterns, Performance, and Flexibility in Elite Soccer Players. Int. J. Sports Physiol. Perform..

[42] Campa F., Silva A.M., Talluri J., Matias C.N., Badicu G., Toselli S. (2020). Somatotype and Bioimpedance Vector Analysis: A New Target Zone for Male Athletes. Sustainability.

[43] Francavilla V.C., Bongiovanni T., Genovesi F., Minafra P., Francavilla G. (2015). Localized bioelectrical impedance analysis: How useful is it in the follow-up of muscle injury? A case report. Med. Dello Sport.

[44] Levi Micheli M., Cannataro R., Gulisano M., Mascherini G. (2022). Proposal of a New Parameter for Evaluating Muscle Mass in Footballers through Bioimpedance Analysis. Biology.

[45] Mascherini G., Castizo-Olier J., Irurtia A., Petri C., Galanti G. (2017). Differences between the sexes in athletes’ body composition and lower limb bioimpedance values. Muscles Ligaments Tendons J..

[46] Mascherini G., Gatterer H., Lukaski H., Burtscher M., Galanti G. (2015). Changes in hydration, body-cell mass and endurance performance of professional soccer players through a competitive season. J. Sports Med. Phys. Fit..

[47] Mascherini G., Petri C., Galanti G. (2015). Integrated total body composition and localized fat-free mass assessment. Sport Sci. Health.

[48] Mascherini G., Petri C., Galanti G. (2019). Link between body cellular mass and left ventricular hypertrophy in female and male athletes. J. Sports Med. Phys. Fit..

[49] Moya-Amaya H., Molina-López A., Berralaguilar A.J., Rojano-Ortega D., La Rosa C.J.B.-D., La Rosa F.J.B.-D. (2021). Bioelectrical Phase Angle, Muscle Damage Markers and Inflammatory Response After a Competitive Match in Professional Soccer Players. Pol. J. Sport Tour..

[50] Petri C., Mascherini G., Pengue L., Galanti G. (2016). Dietary habits in elite soccer players. Sport Sci. Health.

[51] Suarez-Arrones L., Petri C., Maldonado R.A., Torreno N., Munguía-Izquierdo D., Di Salvo V., Méndez-Villanueva A. (2018). Body fat assessment in elite soccer players: Cross-validation of different field methods. Sci. Med. Footb..

[52] Martinez-Ferran M., Rafei E., Romero-Morales C., Pérez-Ruiz M., Lam-Meléndez A., Munguia-Izquierdo D., Pareja-Galeano H. (2022). Optimizing Field Body Fat Percentage Assessment in Professional Soccer Players. Appl. Sci..

[53] Munguia-Izquierdo D., Suarez-Arrones L., Di Salvo V., Paredes-Hernandez V., Alcazar J., Ara I., Kreider R., Mendez-Villanueva A. (2018). Validation of field methods to assess body fat percentage in elite youth soccer players. Int. J. Sports Med..

[54] Munguía-Izquierdo D., Suárez-Arrones L., Di Salvo V., Paredes-Hernández V., Ara I., Mendez-Villanueva A. (2019). Estimating fat-free mass in elite youth male soccer players: Cross-validation of different field methods and development of prediction equation. J. Sports Sci..

[55] Nescolarde L., Terricabras J., Mechó S., Rodas G., Yanguas J. (2020). Differentiation Between Tendinous, Myotendinous and Myofascial Injuries by L-BIA in Professional Football Players. Front. Physiol..

[56] Nescolarde L., Yanguas J., Lukaski H., Alomar X., Rosell-Ferrer J., Rodas G. (2014). Effects of muscle injury severity on localized bioimpedance measurements. Physiol. Meas..

[57] Nescolarde L., Yanguas J., Terricabras J., Lukaski H., Alomar X., Rosell-Ferrer J., Rodas G. (2017). Detection of muscle gap by L-BIA in muscle injuries: Clinical prognosis. Physiol. Meas..

[58] Núñez F.J., Munguía-Izquierdo D., Suárez-Arrones L. (2020). Validity of Field Methods to Estimate Fat-Free Mass Changes Throughout the Season in Elite Youth Soccer Players. Front. Physiol..

[59] Tornero-Aguilera J.F., Villegas-Mora B.E., Clemente-Suárez V.J. (2022). Differences in Body Composition Analysis by DEXA, Skinfold and BIA Methods in Young Football Players. Children.

[60] Fernandes R., Martins A.D., Clemente F.M., Brito J.P., Nobari H., Reis V., Oliveira R. (2025). Variations of distance and accelerometry-based GPS measures and their influence on body composition in professional women soccer players. Proc. Inst. Mech. Eng.-Part P-J. Sports Eng. Technol..

[61] Leão C., Simões M., Silva B., Clemente F.M., Bezerra P., Camões M. (2017). Body Composition Evaluation Issue among Young Elite Football Players: DXA Assessment. Sports.

[62] Oliveira R., Brito J.P., Fernandes R., Morgans R., Alves S., Santos F.J., Pinto P., Espada M.C. (2023). The Effects of Pre-Season and Relationships with Physical, Physiological, Body Composition, and Load Markers: A Case Study Comparing Starters versus Non-Starters from an Elite Female Professional Soccer Team. Medicina.

[63] Oliveira R., Francisco R., Fernandes R., Martins A., Nobari H., Clemente F.M., Brito J.P. (2021). In-season body composition effects in professional women soccer players. Int. J. Environ. Res. Public Health.

[64] Nabuco H.C.G., Silva A.M., Sardinha L.B., Rodrigues F.B., Tomeleri C.M., Ravagnani F.C.P., Cyrino E.S., Ravagnani C.F.C. (2019). Phase Angle is Moderately Associated with Short-term Maximal Intensity Efforts in Soccer Players. Int. J. Sports Med..

[65] Gatterer H., Schenk K., Ferrari P., Faulhaber M., Schopp E., Burtscher M. (2011). Changes in hydration status of soccer players competing in the 2008 European Championship. J. Sports Med. Phys. Fit..

[66] Yargic M.P., Kurklu G.B., Celen M.C., Goktepe E. (2020). Seasonal body composition alterations of an elite male soccer team evaluated with skinfold thickness equations and BIMP analysis. Comp. Exerc. Physiol..

[67] Campa F., Thomas D.M., Watts K., Clark N., Baller D., Morin T., Toselli S., Koury J.C., Melchiorri G., Andreoli A. (2022). Reference Percentiles for Bioelectrical Phase Angle in Athletes. Biology.

[68] Sebastiá-Rico J., Soriano J.M., González-Gálvez N., Martínez-Sanz J.M. (2023). Body Composition of Male Professional Soccer Players Using Different Measurement Methods: A Systematic Review and Meta-Analysis. Nutrients.

[69] Mujika I., Padilla S. (2000). Detraining: Loss of training-induced physiological and performance adaptations. Part I: Short term insufficient training stimulus. Sports Med..

[70] Silva J.R., Brito J., Akenhead R., Nassis G.P. (2016). The Transition Period in Soccer: A Window of Opportunity. Sports Med..

[71] Suarez-Arrones L., Lara-Lopez P., Maldonado R., Torreno N., De Hoyo M., Nakamura F.Y., Di Salvo V., Mendez-Villanueva A. (2019). The effects of detraining and retraining periods on fat-mass and fat-free mass in elite male soccer players. PeerJ.

[72] Ostojic S.M. (2003). Seasonal alterations in body composition and sprint performance of elite soccer players. J. Exerc. Physiol..

[73] Caldwell B.P., Peters D.M. (2009). Seasonal Variation in Physiological Fitness of a Semiprofessional Soccer Team. J. Strength Cond. Res..

[74] Clemente F.M., Ramirez-Campillo R., Sarmento H. (2021). Detrimental Effects of the Off-Season in Soccer Players: A Systematic Review and Meta-analysis. Sports Med..

[75] Martins F., França C., Henriques R., Ihle A., Przednowek K., Marques A., Lopes H., Sarmento H., Gouveia É.R. (2022). Body composition variations between injured and non-injured professional soccer players. Sci. Rep..

[76] McEwan G.P., Drobnic F., Lizarraga A., Gómez Díaz A., Pons E., Dello Iacon A., Unnithan V. (2020). Changes in markers of body composition of professional male soccer players during pre-season. Sports Med. Health Sci..

[77] Milanese C., Cavedon V., Corradini G., De Vita F., Zancanaro C. (2015). Seasonal DXA-measured body composition changes in professional male soccer players. J. Sports Sci..

[78] Campa F., Coratella G., Cerullo G., Noriega Z., Francisco R., Charrier D., Irurtia A., Lukaski H., Silva A.M., Paoli A. (2024). High-standard predictive equations for estimating body composition using bioelectrical impedance analysis: A systematic review. J. Transl. Med..

[79] Matias C.N., Campa F., Santos D.A., Lukaski H., Sardinha L.B., Silva A.M. (2021). Fat-free Mass Bioelectrical Impedance Analysis Predictive Equation for Athletes using a 4-Compartment Model. Int. J. Sports Med..

[80] O’brien C., Young A., Sawka M. (2002). Bioelectrical impedance to estimate changes in hydration status. Int. J. Sports Med..

[81] Ward L.C. (2019). Bioelectrical impedance analysis for body composition assessment: Reflections on accuracy, clinical utility, and standardisation. Eur. J. Clin. Nutr..

[82] Collins J., Maughan R.J., Gleeson M., Bilsborough J., Jeukendrup A., Morton J.P., Phillips S.M., Armstrong L., Burke L.M., Close G.L. (2021). UEFA expert group statement on nutrition in elite football. Current evidence to inform practical recommendations and guide future research. Br. J. Sports Med..

[83] Bennett J.P., Cataldi D., Liu Y.E., Kelly N.N., Quon B.K., Gonzalez M.C., Heymsfield S.B., Shepherd J.A. (2024). Variations in bioelectrical impedance devices impact raw measures comparisons and subsequent prediction of body composition using recommended estimation equations. Clin. Nutr. ESPEN.

[84] Matias C.N., Santos D.A., Júdice P.B., Magalhães J.P., Minderico C.S., Fields D.A., Lukaski H.C., Sardinha L.B., Silva A.M. (2016). Estimation of total body water and extracellular water with bioimpedance in athletes: A need for athlete-specific prediction models. Clin. Nutr..

[85] Matthews E.L., Hosick P.A. (2019). Bioelectrical impedance analysis does not detect an increase in total body water following isotonic fluid consumption. Appl. Physiol. Nutr. Metab..

[86] Francisco R., Jesus F., Gomes T., Nunes C.L., Rocha P., Minderico C.S., Heymsfield S.B., Lukaski H., Sardinha L.B., Silva A.M. (2021). Validity of water compartments estimated using bioimpedance spectroscopy in athletes differing in hydration status. Scand. J. Med. Sci. Sports.

[87] Maughan R.J., Leiper J.B. (1994). Fluid replacement requirements in soccer. J. Sports Sci..

[88] Cutrufello P.T., Dixon C.B., Zavorsky G.S. (2016). Hydration assessment among marathoners using urine specific gravity and bioelectrical impedance analysis. Res. Sports Med..

[89] Baumgartner R.N., Ross R., Heymsfield S.B. (1998). Does adipose tissue influence bioelectric impedance in obese men and women?. J. Appl. Physiol..

[90] Camina Martín M.A., de Mateo Silleras B., Nescolarde Selva L., Barrera Ortega S., Domínguez Rodríguez L., Redondo del Río M.P. (2015). Bioimpedance vector analysis and conventional bioimpedance to assess body composition in older adults with dementia. Nutrition.

[91] Marini E., Sergi G., Succa V., Saragat B., Sarti S., Coin A., Manzato E., Buffa R. (2013). Efficacy of specific bioelectrical impedance vector analysis (BIVA) for assessing body composition in the elderly. J. Nutr. Health Aging.

[92] Buffa R., Saragat B., Cabras S., Rinaldi A.C., Marini E. (2013). Accuracy of specific BIVA for the assessment of body composition in the United States population. PLoS ONE.

[93] Toselli S., Marini E., Maietta Latessa P., Benedetti L., Campa F. (2020). Maturity Related Differences in Body Composition Assessed by Classic and Specific Bioimpedance Vector Analysis among Male Elite Youth Soccer Players. Int. J. Environ. Res. Public Health.

[94] Martins P.C., Gobbo L.A., Silva D.A.S. (2021). Bioelectrical impedance vector analysis (BIVA) in university athletes. J. Int. Soc. Sports Nutr..

[95] Martins P.C., Hansen F., Silva A.M., Silva D.A.S. (2019). Fluid distribution and cell integrity indicators evaluated by bioelectrical impedance in university athletes: Comparison between team sports and individual sports. Physiol. Meas..

[96] Campa F., Toselli S. (2018). Bioimpedance vector analysis of elite, subelite, and low-level male volleyball players. Int. J. Sports Physiol. Perform..

[97] Sutton L., Scott M., Wallace J., Reilly T. (2009). Body composition of English Premier League soccer players: Influence of playing position, international status, and ethnicity. J. Sports Sci..

[98] Piccoli A., Nigrelli S., Caberlotto A., Bottazzo S., Rossi B., Pillon L., Maggiore Q. (1995). Bivariate normal values of the bioelectrical impedance vector in adult and elderly populations. Am. J. Clin. Nutr..

[99] Campa F., Matias C., Gatterer H., Toselli S., Koury J.C., Andreoli A., Melchiorri G., Sardinha L.B., Silva A.M. (2019). Classic bioelectrical impedance vector reference values for assessing body composition in male and female athletes. Int. J. Environ. Res. Public Health.

[100] Bongiovanni T., Rossi A., Trecroci A., Martera G., Iaia F.M., Alberti G., Pasta G., Lacome M. (2022). Regional bioelectrical phase angle is more informative than whole-body phase angle for monitoring neuromuscular performance: A pilot study in elite young soccer players. Sports.

[101] Thomas D.T., Erdman K.A., Burke L.M. (2016). Nutrition and athletic performance. Med. Sci. Sports Exerc..

[102] Chapelle L., Tassignon B., Rommers N., Mertens E., Mullie P., Clarys P. (2020). Pre-exercise hypohydration prevalence in soccer players: A quantitative systematic review. Eur. J. Sport Sci..

[103] Gatterer H., Schenk K., Laninschegg L., Schlemmer P., Lukaski H., Burtscher M. (2014). Bioimpedance Identifies Body Fluid Loss after Exercise in the Heat: A Pilot Study with Body Cooling. PLoS ONE.

[104] Silva A.M., Matias C.N., Nunes C.L., Santos D.A., Marini E., Lukaski H.C., Sardinha L.B. (2019). Lack of agreement of in vivo raw bioimpedance measurements obtained from two single and multi-frequency bioelectrical impedance devices. Eur. J. Clin. Nutr..

[105] Nescolarde L., Lukaski H., De Lorenzo A., de-Mateo-Silleras B., Redondo-del-Río M.P., Camina-Martín M.A. (2016). Different displacement of bioimpedance vector due to Ag/AgCl electrode effect. Eur. J. Clin. Nutr..

[106] Lukaski H.C. (2019). Letter to the Editor: Normal Reference Plots of the Bioelectrical Impedance Vector for Healthy Korean Adults. J. Korean Med. Sci..

[107] Akamatsu Y., Kusakabe T., Arai H., Yamamoto Y., Nakao K., Ikeue K., Ishihara Y., Tagami T., Yasoda A., Ishii K. (2022). Phase angle from bioelectrical impedance analysis is a useful indicator of muscle quality. J. Cachexia Sarcopenia Muscle.

[108] Hetherington-Rauth M., Leu C.G., Júdice P.B., Correia I.R., Magalhães J.P., Sardinha L.B. (2021). Whole body and regional phase angle as indicators of muscular performance in athletes. Eur. J. Sport Sci..

[109] Mattiello R., Amaral M.A., Mundstock E., Ziegelmann P.K. (2020). Reference values for the phase angle of the electrical bioimpedance: Systematic review and meta-analysis involving more than 250,000 subjects. Clin. Nutr..

[110] Martins P.C., Teixeira A.S., Guglielmo L.G.A., Francisco J.S., Silva D.A.S., Nakamura F.Y., Lima L.R.A. (2021). Phase Angle Is Related to 10 m and 30 m Sprint Time and Repeated-Sprint Ability in Young Male Soccer Players. Int. J. Environ. Res. Public Health.

[111] Yamaguchi S., Inami T., Ishida H., Nagata N., Murayama M., Morito A., Yamada S., Kohtake N. (2024). Bioimpedance analysis for identifying new indicators of exercise-induced muscle damage. Sci. Rep..

[112] Pérez-Castillo Í.M., Rueda R., Bouzamondo H., López-Chicharro J., Mihic N. (2023). Biomarkers of post-match recovery in semi-professional and professional football (soccer). Front. Physiol..

[113] Nescolarde L., Talluri A., Yanguas J., Lukaski H. (2023). Phase angle in localized bioimpedance measurements to assess and monitor muscle injury. Rev. Endocr. Metab. Disord..

[114] Stratton M.T., Smith R.W., Harty P.S., Rodriguez C., Johnson B.A., Dellinger J.R., Williams A.D., White S.J., Benavides M.L., Tinsley G.M. (2021). Longitudinal agreement of four bioimpedance analyzers for detecting changes in raw bioimpedance during purposeful weight gain with resistance training. Eur. J. Clin. Nutr..

[115] Siedler M.R., Rodriguez C., Stratton M.T., Harty P.S., Keith D.S., Green J.J., Boykin J.R., White S.J., Williams A.D., DeHaven B. (2023). Assessing the reliability and cross-sectional and longitudinal validity of fifteen bioelectrical impedance analysis devices. Br. J. Nutr..

[116] Siedler M.R., Harty P.S., Stratton M.T., Rodriguez C., Keith D., Green J., Boykin J., Dellinger J., White S., Williams A.D. Day-to-day precision error and least significant change for two commonly used bioelectrical impedance analysis devices. Proceedings of the International Journal of Exercise Science: Conference Proceedings.

[117] Caton J.R., Molé P.A., Adams W.C., Heustis D.S. (1988). Body composition analysis by bioelectrical impedance: Effect of skin temperature. Med. Sci. Sports Exerc..

