# Supplementary material for: Bioelectrical Impedance Analysis in Professional and Semi-Professional Football: A Scoping Review
Source: Sports (Basel). 2025 Oct 3;13(10):348. doi: 10.3390/sports13100348 (PMC12568126; doi:10.3390/sports13100348)
Supplement: Supplementary file 1 [file sports-13-00348-s001.zip › sports-3865728-supplementary/sports-3865728-supplementary v5/Supplementary_2___Search_Strategy.pdf]

Supplementary 2

Preliminary search (Mar 26th, 2025)

"scoping" AND ("soccer" OR "football") AND ("bioelectrical" OR "impedance" OR "impedance" OR "BIA" OR "phase angle" OR "PhA")

Table S1 – Full search strategy

Database	Search String	Nº of records retrieved	Data of search
PubMed/MEDLINE (Title/Abstract)	("soccer"[Title/Abstract] OR "football"[Title/Abstract] OR "sport*"[Title/Abstract] OR "exercis*"[Title/Abstract]) AND ("bioelectrical"[Title/Abstract] OR "bioimpedance"[Title/Abstract] OR "impedance"[Title/Abstract] OR "BIA"[Title/Abstract] OR "phase angle"[Title/Abstract] OR "PhA"[Title/Abstract] OR "reactance"[Title/Abstract] OR "electrical resistance"[Title/Abstract])	2,932	Mar 28th, 2025
EMBASE (Title/Abstract/Keywords)	('soccer'/exp OR 'soccer' OR 'football'/exp OR 'football' OR 'sport*' OR 'exercis*') AND ('bioelectrical':ab,kw,ti OR 'bioimpedance':ab,kw,ti OR 'impedance':ab,kw,ti OR 'bia':ab,kw,ti OR 'phase angle':ab,kw,ti OR 'pha':ab,kw,ti OR 'reactance':ab,kw,ti OR 'electrical resistance':ab,kw,ti) AND [embase]/lim	5,603	Mar 28th, 2025
Web of Science Core Collection (Topic)	("soccer" OR "football" OR "sport*" OR "exercis*") AND ("bioelectrical" OR "bioimpedance" OR "impedance" OR "BIA" OR "phase angle" OR "PhA" OR "reactance" OR "electrical resistance")	5,098	Mar 28th, 2025
SPORT Discus (Title and abstract)	("soccer" OR "football" OR "sport*" OR "exercis*") AND ("bioelectrical" OR "bioimpedance" OR "impedance" OR "BIA" OR "phase angle" OR "PhA" OR "reactance" OR "electrical resistance")	991	Mar 28th, 2025
