# Supplementary material for: Bioelectrical Impedance Analysis in Professional and Semi-Professional Football: A Scoping Review
Source: Sports (Basel). 2025 Oct 3;13(10):348. doi: 10.3390/sports13100348 (PMC12568126; doi:10.3390/sports13100348)
Supplement: Supplementary file 1 [file sports-13-00348-s001.zip › sports-3865728-supplementary/sports-3865728-supplementary v5/Supplementary_3___Non_retrieved_and_Excluded_Records.pdf]

**Supplementary 3**

**Table S2 – Non-retrieved records, and records excluded after full-text reading**

| Reference | Reason |
|---|---|
| Ahsan et al., 2023 [1] | Only reports quantitative BIA-estimated body composition outcomes without comparison with or validation against other techniques |
| Alvero-Cruz et al., 2017 [2] | Not available in English |
| Andreoli et al., 2003 [3] | Only reports quantitative BIA-estimated body composition outcomes without comparison with or validation against other techniques |
| Argüello et a., 2020 [4] | Abstract only / Conference communication |
| Bandyopadhyay et al., 2018 [5] | Only reports quantitative BIA-estimated body composition outcomes without comparison with or validation against other techniques |
| Bandyopadhyay et al., 2016 [6] | Abstract only / Conference communication |
| Baranauskas et al., 2020 [7] | No football player was recruited or analyzed |
| Berral-Aguilar et al., 2021 [8] | Not available in English |
| Blanco-Espitia et al., 2023 [9] | Not available in English |
| Bongiovanni et a., 2023 [10] | Only reports quantitative BIA-estimated body composition outcomes without comparison with or validation against other techniques |
| Bonuccelli et al., 2011 [11] | Abstract only / Conference communication |
| Boraczyński et al ., 2012 [12] | Unclear competitive level |
| Burdukiewicz et al., 2013 [13] | Only reports quantitative BIA-estimated body composition outcomes without comparison with or validation against other techniques |
| Caldwell & Peters 2009 [14] | Only reports quantitative BIA-estimated body composition outcomes without comparison with or validation against other techniques |
| Campa et al., 2021 [15] | Different context |
| Campa et al., 2020 [16] | No separated analysis was conducted in football players |
| Campa et al., 2020 [17] | No football player was recruited or analyzed |
| Castillo-Gonzalez et al., 2023 [18] | Not available in English |
| Cattem et al., 2024 [19] | <16 years old |
| Causevic et al., 2024 [20] | Only reports quantitative BIA-estimated body composition outcomes without comparison with or validation against other techniques |
| Čolakhodžić et al., 2010 [21] | Only reports quantitative BIA-estimated body composition outcomes without comparison with or validation against other techniques |
| Čolakhodžić et al., 2011 [22] | Unclear competitive level |
| Dagcilar et al., 2020 [23] | Only reports quantitative BIA-estimated body composition outcomes without comparison with or validation against other techniques |
| Dimitrijevic et al., 2024 [24] | No separated analysis was conducted in football players |
| Dobrowolski & Włodarek, 2020 [25] | Only reports quantitative BIA-estimated body composition outcomes without comparison with or validation against other techniques |
| Dobrowolski & Włodarek, 2023 [26] | Only reports quantitative BIA-estimated body composition outcomes without comparison with or validation against other techniques |
| Eraslan et al., 2025 [27] | Evaluates the effect of a non-routine intervention |
| Francisco et al., 2020 [28] | No separated analysis was conducted in football players |
| Galanti et al., 2015 [29] | <16 years old |
| Gatteschi et al., 2011 [30] | Abstract only / Conference communication |
| Ghiani et a., 2022 [31] | Different context |
| Gómez-Figueroa et al., 2023 [32] | Not available in English |
| González et al., 2023 [33] | Not available in English |
| Hetherington-Rauth et al., 2021 [34] | No separated analysis was conducted in football players |
| Ishiguro et al., 2005 [35] | Unclear competitive level |
| Jimenez et al., 2020 [36] | Abstract only / Conference communication |

| | |
|---|---|
| Jorn et al., 2019 [37] | Abstract only / Conference communication |
| Kenger et al., 2023 [38] | Only reports quantitative BIA-estimated body composition outcomes without comparison with or validation against other techniques |
| Komiya et al., 2012 [39] | Not retrievable |
| Koury et al., 2014 [40] | <16 years old |
| Kr Dey et al., 2015 [41] | <16 years old |
| Küçük & Söyler 2024 [42] | No BIA assessment |
| Küçükkubaş et al., 2019 [43] | <16 years old |
| Kutáč 2012 [44] | Only reports quantitative BIA-estimated body composition outcomes without comparison with or validation against other techniques |
| Kutáč 2013 [45] | Only reports quantitative BIA-estimated body composition outcomes without comparison with or validation against other techniques |
| Kutáč 2014 [46] | Only reports quantitative BIA-estimated body composition outcomes without comparison with or validation against other techniques |
| Lai et al., 2024 [47] | No separated analysis was conducted in football players |
| Lombardi et al., 2021 [48] | Not available in English |
| Lukaski et al., 1990 [49] | No separated analysis was conducted in football players |
| Macuh et al., 2023 [50] | Only reports quantitative BIA-estimated body composition outcomes without comparison with or validation against other techniques |
| Madic et al., 2018 [51] | <16 years old |
| Mala et al., 2015 [52] | Only reports quantitative BIA-estimated body composition outcomes without comparison with or validation against other techniques |
| Mala et al., 2020 [53] | Only reports quantitative BIA-estimated body composition outcomes without comparison with or validation against other techniques |
| Mala et al., 2023 [54] | Only reports quantitative BIA-estimated body composition outcomes without comparison with or validation against other techniques |
| Marini et al., 2020 [55] | No separated analysis was conducted in football players |
| Marra et al., 2021 [56] | No football player was recruited or analyzed |
| Marra et al., 1996 [57] | Not retrievable |
| Martins et al., 2021 [58] | <16 years old |
| Mascherini et al., 2014 [59] | Abstract only / Conference communication |
| Mascherini et al., 2014 [60] | Abstract only / Conference communication |
| Matias et al., 2016 [61] | No separated analysis was conducted in football players |
| Matias et al., 2019 [62] | No football player was recruited or analyzed |
| Matias et al., 2016 [63] | No separated analysis was conducted in football players |
| Matias et al., 2015 [64] | Not retrievable |
| Mauro-Martin et al., 2015 [65] | Not available in English |
| Melchiorri et al., 2000 [66] | Not retrievable |
| Melchiorri et al., 2007 [67] | Only reports quantitative BIA-estimated body composition outcomes without comparison with or validation against other techniques |
| MetikoŠ et al., 2014 [68] | No separated analysis was conducted in football players |
| Micheli et al., 2011 [69] | Unclear competitive level |
| Molina-López et al., 2022 [70] | Not available in English |
| Nescolarde et al., 2011 [71] | Abstract only / Conference communication |
| Nescolarde et al., 2014 [72] | Abstract only / Conference communication |
| Ostoiic et al., 2006 [73] | Not retrievable |
| Oukheda et al., 2024 [74] | Only reports quantitative BIA-estimated body composition outcomes without comparison with or validation against other techniques |
| Prada et al., 2017 [75] | Abstract only / Conference communication |
| Rosa et al., 2022 [76] | Unclear competitive level |
| Santos et al., 2015 [77] | No separated analysis was conducted in football players |
| Sardinha et al., 2020 [78] | No separated analysis was conducted in football players |

| | |
|---|---|
| Sarkar et al., 2019 [79] | <16 years old |
| Sganzerla et al., 2025 [80] | Unclear competitive level |
| Silva et al., 2019 [81] | No separated analysis was conducted in football players |
| Skorupan et al., 2011[82] | Abstract only / Conference communication |
| Spehnjak et al., 2021 [83] | Only reports quantitative BIA-estimated body composition outcomes without comparison with or validation against other techniques |
| Staśkiewicz et al., 2022 [84] | Only reports quantitative BIA-estimated body composition outcomes without comparison with or validation against other techniques |
| Staśkiewicz et al., 2022 [85] | Only reports quantitative BIA-estimated body composition outcomes without comparison with or validation against other techniques |
| Staśkiewicz et al., 2023 [86] | Only reports quantitative BIA-estimated body composition outcomes without comparison with or validation against other techniques |
| Staśkiewicz et al., 2023 [87] | Only reports quantitative BIA-estimated body composition outcomes without comparison with or validation against other techniques |
| Staśkiewicz et al., 2024 [88] | Only reports quantitative BIA-estimated body composition outcomes without comparison with or validation against other techniques |
| Stewart and Hannan [89] | No football player was recruited or analyzed |
| Tereso et al., 2021 [90] | Only reports quantitative BIA-estimated body composition outcomes without comparison with or validation against other techniques |
| Tereso et al., 2024 [91] | Not available in English |
| Torres-Luque et al., 2015 [92] | <16 years old |
| Toskic et al., 2024 [93] | No separated analysis was conducted in football players |
| Ushiki et al., 2023 [94] | Only reports quantitative BIA-estimated body composition outcomes without comparison with or validation against other techniques |
| Vici et al., 2019 [95] | Abstract only / Conference communication |
| Yamaguchi et al., 2024 [96] | No football player was recruited or analyzed |
| Zeleznik et al., 2023 [97] | Only reports quantitative BIA-estimated body composition outcomes without comparison with or validation against other techniques |
| Zombra 2019 [98] | Only reports quantitative BIA-estimated body composition outcomes without comparison with or validation against other techniques |

## References


1. Ahsan M, Ali MF. A comparative evaluation of anthropometric characteristics and respiratory functions' parameters among rugby and soccer players. Physical Activity Review. 2023;11(1):31-9.
2. Alvero-Cruz JR, Ronconi M, Romero JCG, Gill MCD, López MJ, Gómez LC, et al. Body composition changes after sport detraining period. NUTRICION HOSPITALARIA. 2017;34(3):632-8. doi: 10.20960/nh.618.
3. Andreoli A, Melchiorri G, Brozzi M, Di Marco A, Volpe SL, Garofano P, et al. Effect of different sports on body cell mass in highly trained athletes. Acta Diabetologica. 2003;40(SUPPL. 1):S122-S5. doi: 10.1007/s00592-003-0043-9.
4. Argüello P, Gálvez A, Castro L, Sánchez I, Melo P. The phase angle as a marker of health and body composition in soccer players in Bogota - Colombia. EUROPEAN JOURNAL OF PUBLIC HEALTH. 2020;30.
5. Bandyopadhyay A, Chatterjee, Jana S, Dey SK. Assessment of body water spaces & mineral content in trained athletes of different sports using bioelectrical impedance analysis. Baltic Journal of Health & Physical Activity. 2018;10(2):43-54.

6. Bandyopadhyay A, Chatterjee S, Datta G, Dey SK. Effect of intracellular hydration on isometric strength & aerobic capacity in different age groups of elite young male athletes playing different sports. Indian Journal of Physiology and Pharmacology. 2016;60(5):42-3.
7. Baranauskas M, Jablonskienė V, Abaravičius JA, Stukas R. Actual Nutrition and Dietary Supplementation in Lithuanian Elite Athletes. Medicina (Kaunas). 2020;56(5). doi: 10.3390/medicina56050247.
8. Berral-Aguilar AJ, Mendez-Rebolledo G, Rojano-Ortega D, Moya-Amaya H, Molina-López A, Berral-De-la-Rosa FJ. Assessment of the Impact of Confinement by SARS-CoV-2 on the Body Composition of Elite Soccer Players. INTERNATIONAL JOURNAL OF MORPHOLOGY. 2021;39(4):1088-95.
9. Blanco-Espitia DC, Blanco-Espitia RD, Gálvez-Pardo AY, Argüello-Gutiérrez YP, Castro-Jimenez LE. Medición del consumo máximo de oxígeno en futbolistas profesionales de Bogotá. Actividad Fisica & Deporte. 2023;9(1):1-6.
10. Bongiovanni T, Rossi A, Genovesi F, Martera G, Puleo G, Orlandi C, et al. How Do Football Playing Positions Differ in Body Composition? A First Insight into White Italian Serie A and Serie B Players. Journal of Functional Morphology and Kinesiology. 2023;8(2):80.
11. Bonuccelli A, Marzatico F, Stesina G, Stefanini L, Buonocore D, Rucci S, et al. Bioelectrical impedance vector analysis (BIVA) to evaluate seasonal variations in body composition of elite soccer players. Journal of the International Society of Sports Nutrition. 2011;8(Suppl 1):1-.
12. Boraczyński T, Boraczyński M, Obmiński Z, Stasiewicz P, Podstawski R, Stasiewicz K, et al. BUDOWA SOMATYCZNA I WYDOLNOŚĆ FIZYCZNA PIŁKARZY NOŻNYCH I SIATKARZY W WIEKU 16-18 LAT. Polish Journal of Sports Medicine / Medycyna Sportowa. 2012;28(1):39-49.
13. Burdukiewicz A, Chmura JAN, Pietraszewska J, Andrzejewska J, Stachon A, Nosal J. CHARACTERISTICS OF BODY TISSUE COMPOSITION AND FUNCTIONAL TRAITS IN JUNIOR FOOTBALL PLAYERS. Human Movement. 2013;14(2):96-101.
14. Caldwell BP, Peters DM. Seasonal variation in physiological fitness of a semiprofessional soccer team. Journal of strength and conditioning research. 2009;23(5):1370-7. doi: 10.1519/JSC.0b013e3181a4e82f.
15. Campa F, Bongiovanni T, Trecroci A, Rossi A, Greco G, Pasta G, et al. Effects of the COVID-19 Lockdown on Body Composition and Bioelectrical Phase Angle in Serie A Soccer Players: A Comparison of Two Consecutive Seasons. Biology (Basel). 2021;10(11). doi: 10.3390/biology10111175.
16. Campa F, Silva AM, Matias CN, Monteiro CP, Paoli A, Nunes JP, et al. Body water content and morphological characteristics modify bioimpedance vector patterns in volleyball, soccer, and rugby players. International Journal of Environmental Research and Public Health. 2020;17(18):1-12. doi: 10.3390/ijerph17186604.
17. Campa F, Matias CN, Marini E, Heymsfield SB, Toselli S, Sardinha LB, et al. Identifying Athlete Body Fluid Changes During a Competitive Season With Bioelectrical Impedance Vector Analysis. International journal of sports physiology and performance. 2020;15(3):361-7. doi: 10.1123/ijspp.2019-0285.
18. Castillo González WN, Soriano Castañeda SF, Rodríguez Prieto IE. Composición corporal y aptitud física en las divisiones menores de un equipo de fútbol profesional colombiano. Retos: Nuevas Perspectivas de Educación Física, Deporte y Recreación. 2023;48:271-6.

19. Cattem MVDO, Coelho GMDO, Koury JC. Fat-free mass predictive equation using multifrequency bioelectrical impedance data in adolescent soccer athletes: development and cross-validation. Nutrition. 2024;125. doi: 10.1016/j.nut.2024.112484.
20. Causevic D, Mustafovic E, Rani B, Klacar S, Ibrahimovic M, Panaet AE, et al. Effects of 5-week detraining period on body composition of professional soccer players. Health Sport Rehabilitation. 2024;10(2):47-58.
21. Čolakhodžić E, Vidović N, Fazlagić S. Changes in body structure of adult football players during one training unit. Homo Sporticus. 2010;12(1):51-6.
22. Čolakhodžić E, Popo A, Bajramović I, Likić S. Trend indicators of changes in body composition in soccer players in different periods of their career. Homo Sporticus. 2011;13(1):21-7.
23. Dagcilar K, Öztürk M. An evaluation of nutritional knowledge levels, nutritional intake, and anthropometric features of Northern Cyprus professional football players. MEDICINA DELLO SPORT. 2020;73(1):81-9. doi: 10.23736/S0025-7826.20.03573-5.
24. Dimitrijevic M, Lalovic D, Milovanov D. Correlation of Different Anthropometric Methods and Bioelectric Impedance in Assessing Body Fat Percentage of Professional Male Athletes. Experimental and Applied Biomedical Research (EABR). 2024;25(2):127-36. doi: 10.2478/sjecr-2021-0026.
25. Dobrowolski H, Włodarek D. Low energy availability in group of Polish female soccer players. Rocz Panstw Zakl Hig. 2020;71(1):89-96. doi: 10.32394/rpzh.2020.0106.
26. Dobrowolski H, Włodarek D. Energy expenditure during training and official league match in professional female soccer players - a pilot study. Rocz Panstw Zakl Hig. 2023;74(2):143-50. doi: 10.32394/rpzh.2023.0253.
27. Eraslan M, Gürkan AC, Aydın S, Şahin M, Çelik S, Söyler M, et al. The Effect of Proprioceptive Training on Technical Soccer Skills in Youth Professional Soccer. Medicina (Kaunas). 2025;61(2). doi: 10.3390/medicina61020252.
28. Francisco R, Matias CN, Santos DA, Campa F, Minderico CS, Rocha P, et al. The predictive role of raw bioelectrical impedance parameters in water compartments and fluid distribution assessed by dilution techniques in athletes. International Journal of Environmental Research and Public Health. 2020;17(3). doi: 10.3390/ijerph17030759.
29. Galanti G, Stefani L, Scacciati I, Mascherini G, Buti G, Maffulli N. Eating and nutrition habits in young competitive athletes: a comparison between soccer players and cyclists. Transl Med UniSa. 2015;11:44-7.
30. Gatteschi L, Angelini F, Bonuccelli A, Marzatico F, Rubenni M, Zeppilli P. Fluid balance and body composition analysis in female soccer players effect of the match. Journal of the International Society of Sports Nutrition. 2011;8:1-.
31. Ghiani G, Roberto S, Mura R, Fois F, Scorcu M, Crisafulli A. Body composition changes during the lockdown-restart transition due to the SARS-CoV-2 pandemic in a group of professional football players. J Sports Med Phys Fitness. 2022;62(5):649-53. doi: 10.23736/S0022-4707.21.12894-4.
32. Gómez-Figueroa JA, Molina-Arriola JE, Gutiérrez-Bravo YA, Montufar-Romero JL, Aguilar H. ANÁLISIS DEL SOMATOTIPO DE JUGADORES JUVENILES DE FÚTBOL ASOCIACIÓN. Revista Iberoamericana de Ciencias de la Actividad Física y el Deporte. 2023;12(1):163-75.
33. González WNC, Castañeda SFS, Prieto IER. Body Composition and physical fitness in the youth divisions of a Colombian professional soccer team. RETOS-NUEVAS TENDENCIAS EN EDUCACION FISICA DEPORTE Y RECREACION. 2023(48):271-6.

34. Hetherington-Rauth M, Leu CG, Júdice PB, Correia IR, Magalhaes JP, Sardinha LB. Whole body and regional phase angle as indicators of muscular performance in athletes. EUROPEAN JOURNAL OF SPORT SCIENCE. 2021;21(12):1684-92. doi: 10.1080/17461391.2020.1858971.
35. Ishiguro N, Kanehisa H, Miyatani M, Masuo Y, Fukunaga T. A comparison of three bioelectrical impedance analyses for predicting lean body mass in a population with a large difference in muscularity. European Journal of Applied Physiology. 2005;94(1-2):25-35. doi: 10.1007/s00421-004-1259-2.
36. Jimenez LC, Rojas IS, Gutierrez YA, Pardo AG, Buitrago PM. Relationship of the phase angle with the maximum and explosive force in soccer players from Bogota. EUROPEAN JOURNAL OF PUBLIC HEALTH. 2020;30:V677-V.
37. Jorn L, Gunn J, McCluskey I, Mayhew JL, Brechue WF. Using Skinfolds and Bioelectrical Impedance for Tracking Body Composition across a Soccer Season. MEDICINE AND SCIENCE IN SPORTS AND EXERCISE. 2019;51(6):910-. doi: 10.1249/01.mss.0000563228.61643.70.
38. Kenger EB, Eren F, Ozlu T, Gunes FE. Analysis of microbiota profile and nutritional status in male professional football players. J Sports Med Phys Fitness. 2023;63(11):1235-43. doi: 10.23736/S0022-4707.23.15103-6.
39. Komiya H, Tsubakihara M. Estimation of Thigh Muscle Volume by Multi-Frequency Bioelectrical Impedance. Advances in Exercise & Sports Physiology. 2012;18(1):17-25.
40. Koury JC, Trugo NMF, Torres AG. Phase Angle and Bioelectrical Impedance Vectors in Adolescent and Adult Male Athletes. International Journal of Sports Physiology & Performance. 2014;9(5):798-804.
41. Kr Dey S, Bandyopadhyay A, Jana S, Chatterjee S. Assessment of body cell mass in Indian junior elite players (male) of different sports using bioelectrical impedance analysis method. Sports Medicine Journal / Medicina Sportiva. 2015;11(2):2533-40.
42. Küçük H, Söyler M. Body composition, anaerobic power, lower extremity strength in football players: Acute effect on different leagues. Turkish Journal of Kinesiology. 2024;10(1):24-33.
43. Küçükkubaş N, Aytar SH, Açlkada C, Hazlr T. Bioelectric impedance analyses for young male athletes: A validation study. Isokinetics and Exercise Science. 2020;28(1):49-58. doi: 10.3233/IES-185209.
44. Kutáč P. APPLICATION OF TYPICAL ERROR OF MEASUREMENT FOR ACCURACY OF MEASUREMENT OF BODY COMPOSITION IN ATHLETES USING THE BIA METHOD. Medicina Sportiva. 2012;16(4):150-4.
45. Kutáč P. SOMATIC PARAMETERS OF 17 YEAR OLD SOCCER PLAYERS IN THE OLDER YOUTH CATEGORY IN RELATION TO SPORTS PERFORMANCE. Acta Universitatis Palackianae Olomucensis Gymnica. 2013;43(3):17-26.
46. Kutáč P. The effect of intake of water on the final values of body composition parameters in active athletes using two different bioimpedance analyzers. Acta Gymnica. 2014;44(2):107-16.
47. Lai Y-K, Ho C-Y, Huang A-C, Lu H-K, Hsieh K-C. Estimation equation of limb lean soft tissue mass in Asian athletes using bioelectrical impedance analysis. PloS one. 2024;19(8):e0300911. doi: 10.1371/journal.pone.0300911.
48. Lombardi JAB, França EF, Macedo MM, Silva Ad, Reis C. CARACTERIZAÇÃO DA COMPOSIÇÃO CORPORAL DE ATLETAS PROFISSIONAIS DE FUTEBOL QUE DISPUTARAM O CAMPEONATO PAULISTA DE 2018: UMA AVALIAÇÃO BASEADA NA

TÉCNICA DE BIOIMPEDÂNCIA ELÉTRICA. Brazilian Journal of Soccer Science / Revista Brasileira de Futebol. 2021;14(2):19-32.
49. Lukaski HC, Bolonchuk WW, Siders WA, Hall CB. Body composition assessment of athletes using bioelectrical impedance measurements. Journal of Sports Medicine and Physical Fitness. 1990;30(4):434-40.
50. Macuh M, Levec J, Kojić N, Knap B. Dietary Intake, Body Composition and Performance of Professional Football Athletes in Slovenia. Nutrients. 2023;15(1). doi: 10.3390/nu15010082.
51. Madic DM, Andrasic S, Gusic M, Molnar S, Radanovic D, Trajkovic N. Seasonal Body Composition Variations in Adolescent Soccer Players. INTERNATIONAL JOURNAL OF MORPHOLOGY. 2018;36(3):877-80.
52. Mala L, Maly T, Zahalka F, Bunc V, Kaplan A, Jebavy R, et al. Body composition of elite female players in five different sports games. Journal of human kinetics. 2015;45:207.
53. Mala L, Maly T, Cabell L, Hank M, Bujnovsky D, Zahalka F. Anthropometric, body composition, and morphological lower limb asymmetries in elite soccer players: A prospective cohort study. International Journal of Environmental Research and Public Health. 2020;17(4). doi: 10.3390/ijerph17041140.
54. Mala L, Hank M, Stastny P, Zahalka F, Ford KR, Zmijewski P, et al. Elite young soccer players have smaller inter-limb asymmetry and better body composition than non-elite players. Biology of sport. 2023;40(1):265-72. doi: 10.5114/biolsport.2023.114840.
55. Marini E, Campa F, Buffa R, Stagi S, Matias CN, Toselli S, et al. Phase angle and bioelectrical impedance vector analysis in the evaluation of body composition in athletes. Clinical Nutrition. 2020;39(2):447-54. doi: 10.1016/j.clnu.2019.02.016.
56. Marra M, Di Vincenzo O, Cioffi I, Sammarco R, Morlino D, Scalfi L. Resting energy expenditure in elite athletes: development of new predictive equations based on anthropometric variables and bioelectrical impedance analysis derived phase angle. Journal of the International Society of Sports Nutrition. 2021;18(1):68. doi: 10.1186/s12970-021-00465-x.
57. Marra M, Scalfi L, Lanzetta C, DeStasio P, Rosato GF, Contaldo F. Evaluation of body composition in athletes. MEDICINA DELLO SPORT. 1996;49(4):469-75.
58. Martins PC, Teixeira AS, Guglielmo LGA, Francisco JS, Silva DAS, Nakamura FY, et al. Phase Angle Is Related to 10 m and 30 m Sprint Time and Repeated-Sprint Ability in Young Male Soccer Players. International journal of environmental research and public health. 2021;18(9). doi: 10.3390/ijerph18094405.
59. Mascherini G, Laura Stefani L, Gatterer H, Cattozzo A, Galanti G. Bioelectrical impedance and soccer performance in professional soccer players. European Journal of Preventive Cardiology. 2014;21(1):S42. doi: 10.1177/2047487314534575.
60. Mascherini G, Gatterer H, Stefani I, Cattozzo A, Galanti G. The Usefulness Of Bioelectrical Impedance To Monitor The Performance In Professional Soccer Players During A Sport Season. MEDICINE AND SCIENCE IN SPORTS AND EXERCISE. 2014;46(5):851-2. doi: 10.1249/01.mss.0000496054.02396.b5.
61. Matias CN, Júdice PB, Santos DA, Magalhaes JP, Minderico CS, Fields DA, et al. Suitability of Bioelectrical Based Methods to Assess Water Compartments in Recreational and Elite Athletes. JOURNAL OF THE AMERICAN COLLEGE OF NUTRITION. 2016;35(5):413-21.
62. Matias CN, Noujeimi FA, Sardinha LB, Teixeira VH, Silva AM. Total body water and water compartments assessment in athletes: Validity of multi-frequency bioelectrical impedance. Science and Sports. 2019;34(6):e307-e13. doi: 10.1016/j.scispo.2018.11.007.

63. Matias CN, Santos DA, Júdice PB, Magalhães JP, Minderico CS, Fields DA, et al. Estimation of total body water and extracellular water with bioimpedance in athletes: A need for athlete-specific prediction models. Clinical Nutrition. 2016;35(2):468-74. doi: 10.1016/j.clnu.2015.03.013.
64. Matias CN, Santos DA, Júdice PB, Magalhães JP, Minderico CS, Fields DA, et al. Total body and extracellular hydration estimates in highly active adults: Validation of bioelectrical impedance based methods. European Journal of Clinical Nutrition. 2015;69:S34. doi: 10.1038/ejcn.2015.190.
65. Mauro-Martín IS, García-Angulo B, Fajardo D, Garicano-Vilar E. Nutritional and body composition assessment and its relationship with athletic performance in a women's soccer team. Revista Espanola de Nutricion Humana y Dietetica. 2015;19(1):36-48. doi: 10.14306/renhyd.19.1.109.
66. Melchiorri G, Andreoli A, Candeloro N, De Lorenzo A. [Changes in body composition caused by intense physical training]. Clin Ter. 2000;151(2):73-6.
67. Melchiorri G, Monteleone G, Andreoli A, Callà C, Sgroi M, De Lorenzo A. Body cell mass measured by bioelectrical impedance spectroscopy in professional football (soccer) players. The Journal of sports medicine and physical fitness. 2007;47(4):408-12.
68. MetikoŠ B, Kovač S, Čović N, Mekić A. Male Athlete's Body Composition and Postural Balance Correlation. Homo Sporticus. 2014;16(1):5-9.
69. Micheli ML, Gulisano M, Morucci G, Punzi T, Ruggiero M, Ceroti M, et al. Angiotensin-converting enzyme/vitamin D receptor gene polymorphisms and bioelectrical impedance analysis in predicting athletic performances of Italian young soccer players. Journal of strength and conditioning research. 2011;25(8):2084-91. doi: 10.1519/JSC.0b013e31820238aa.
70. Molina-López A, Moya-Amaya H, Estevan-Navarro P, Berral-Aguilar AJ, Rojano-Ortega D, Berral-De la Rosa FJ. Changes in Body Composition and Phase Angle During Pre-Season in Professional Soccer Players. International Journal of Morphology. 2022;40(2):348-54. doi: 10.4067/S0717-95022022000200348.
71. Nescolarde L, Yanguas J, Medina D, Rodas G, Rosell-Ferrer J. Assessment and follow-up of muscle injuries in athletes by bioimpedance: preliminary results. Annual International Conference of the IEEE Engineering in Medicine and Biology Society IEEE Engineering in Medicine and Biology Society Annual International Conference. 2011;2011:1137-40. doi: 10.1109/iembs.2011.6090266.
72. Nescolarde L, Yanguas J, Lukaski H, Rodas G, Rosell-Ferrer J. Localized BIA identifies structural and pathophysiological changes in soft tissue after post-traumatic injuries in soccer players. Annual International Conference of the IEEE Engineering in Medicine and Biology Society IEEE Engineering in Medicine and Biology Society Annual International Conference. 2014;2014:3743-6. doi: 10.1109/embc.2014.6944437.
73. Ostojic SM. Estimation of body fat in athletes: Skinfolds vs bioelectrical impedance. Journal of Sports Medicine and Physical Fitness. 2006;46(3):442-6.
74. Oukheda M, Lebrazi H, Derouiche A, Kettani A, Saile R, Taki H. Performance variables and nutritional status analysis from Moroccan professional and adolescent football players during the competition period: a descriptive study. Frontiers in sports and active living. 2024;6:1372381. doi: 10.3389/fspor.2024.1372381.
75. Prada EO, Longo AF, Lentini NA, Cardey ML, Aquilino GD, Francia ER. Comparison between Anthropometry and Bioelectrical Impedance in Estimating Fat and Muscle Masses in Soccer Players. MEDICINE AND SCIENCE IN SPORTS AND EXERCISE. 2017;49(5):573-. doi: 10.1249/01.mss.0000518490.85006.8d.

76. Rosa F, Sarmento H, Duarte JP, Barrera J, Loureiro F, Vaz V, et al. Knee and hip agonist-antagonist relationship in male under-19 soccer players. PLoS ONE. 2022;17(4 April). doi: 10.1371/journal.pone.0266881.
77. Santos DA, Silva AM, Matias CN, Magalhães JP, Minderico CS, Thomas DM, et al. Utility of novel body indices in predicting fat mass in elite athletes. Nutrition. 2015;31(7-8):948-54. doi: 10.1016/j.nut.2015.02.003.
78. Sardinha LB, Correia IR, Magalhães JP, Júdice PB, Silva AM, Hetherington-Rauth M. Development and validation of BIA prediction equations of upper and lower limb lean soft tissue in athletes. European Journal of Clinical Nutrition. 2020;74(12):1646-52. doi: 10.1038/s41430-020-0666-8.
79. Sarkar S, Chatterjee S, Dey SK. Comparison of body composition, physical fitness parameters and skeletal muscle damage indices among young Indian male soccer and hockey players. Baltic Journal of Health & Physical Activity. 2019;11(2):1-10.
80. Sganzerla G, Gobbo LA, Mendonça ALB, Ravagnani FCDP, Oliveira-Junior SAD, Cordeiro V, et al. Phase angle differences between injured and uninjured athletes: A cross-sectional study. Journal of Bodywork and Movement Therapies. 2025;42:583-8. doi: 10.1016/j.jbmt.2025.01.030.
81. Silva AM, Matias CN, Nunes CL, Santos DA, Marini E, Lukaski HC, et al. Lack of agreement of in vivo raw bioimpedance measurements obtained from two single and multi-frequency bioelectrical impedance devices. European Journal of Clinical Nutrition. 2019;73(7):1077-83. doi: 10.1038/s41430-018-0355-z.
82. Skorupan N, Ivković M. Comparison of anthropometric and cardiovascular adaptive changes in young water polo and soccer players. European Journal of Medical Research. 2011;16:75.
83. Spehnjak M, Gušić M, Molnar S, Baić M, Andrašić S, Selimi M, et al. Body Composition in Elite Soccer Players from Youth to Senior Squad. International journal of environmental research and public health. 2021;18(9). doi: 10.3390/ijerph18094982.
84. Staśkiewicz W, Grochowska-Niedworok E, Zydek G, Białek-Dratwa A, Grajek M, Jaruga-Sękowska S, et al. Changes in body composition during the macrocycle of professional football players in relation to sports nutrition knowledge. Frontiers in nutrition. 2022;9:981894. doi: 10.3389/fnut.2022.981894.
85. StaŚKiewicz W, Grochowska-Niedworok E, Zydek G, Kiciak A, Agnieszka B, Marek K, et al. Changes in body water content of elite soccer players during the macrocycle according to function on the field. Journal of Physical Education & Sport. 2022;22(6):1464-70.
86. Staśkiewicz W, Grochowska-Niedworok E, Zydek G, Grajek M, Krupa-Kotara K, Białek-Dratwa A, et al. The Assessment of Body Composition and Nutritional Awareness of Football Players According to Age. Nutrients. 2023;15(3). doi: 10.3390/nu15030705.
87. Staśkiewicz-Bartecka W, Grochowska-Niedworok E, Zydek G, Grajek M, Kiciak A, Białek-Dratwa A, et al. Anthropometric Profiling and Changes in Segmental Body Composition of Professional Football Players in Relation to Age over the Training Macrocycle. Sports (Basel). 2023;11(9). doi: 10.3390/sports11090172.
88. Staśkiewicz-Bartecka W, Krupa-Kotara K, Rozmiarek M, Malchrowicz-Mośko E, Grajek M, Elordui SA, et al. Anthropometric Profile and Position-Specific Changes in Segmental Body Composition of Professional Football Players Throughout a Training Period. Sports (Basel). 2024;12(10). doi: 10.3390/sports12100285.

89. Stewart AD, Hannan WJ. Prediction of fat and fat-free mass in male athletes using dual X-ray absorptiometry as the reference method. Journal of Sports Sciences. 2000;18(4):263-74. doi: 10.1080/026404100365009.
90. Tereso D, Paulo R, Petrica J, Duarte-Mendes P, Gamonales JM, Ibáñez SJ. Assessment of body composition, lower limbs power, and anaerobic power of senior soccer players in Portugal: Differences according to the competitive level. International Journal of Environmental Research and Public Health. 2021;18(15). doi: 10.3390/ijerph18158069.
91. Tereso D, Gamonales JM, Petrica J, Ibáñez SJ, Paulo R. Avaliação da composição corporal, da potência de membros inferiores e da potência anaeróbia de jogadores de futebol: diferenças consoante a posição em campo. Retos: Nuevas Perspectivas de Educación Física, Deporte y Recreación. 2024;59:1034-45.
92. Torres-Luque G, Calahorro-Cañada F, Lara-Sánchez A, Garatachea N, Nikolaidis P. Body composition using bioelectrical impedance analysis in elite young soccer players: the effects of age and playing position. Sport Sciences for Health. 2015;11(2):203-10.
93. Toskic L, Markovic M, Simenko J, Vidic V, Cikiriz N, Dopsaj M. Analysis of Body Composition in Men and Women with Diverse Training Profiles: A Cross-Sectional Study. International Journal of Morphology. 2024;42(5):1278-87. doi: 10.4067/S0717-95022024000501278.
94. Ushiki T, Mochizuki T, Suzuki K, Kamimura M, Ishiguro H, Suwabe T, et al. Strategic analysis of body composition indices and resting platelet ATP levels in professional soccer players for better platelet-rich plasma therapy. Frontiers in bioengineering and biotechnology. 2023;11:1255860. doi: 10.3389/fbioe.2023.1255860.
95. Vici G, Cesanelli L, Belli L, Vastano G, Polzonetti V. The impact of hydration status on recovery in a soccer team. International Journal of Sport Nutrition and Exercise Metabolism. 2019;29:13. doi: 10.1123/ijsnem.2019-0057.
96. Yamaguchi S, Inami T, Ishida H, Nagata N, Murayama M, Morito A, et al. Bioimpedance analysis for identifying new indicators of exercise-induced muscle damage. Scientific reports. 2024;14(1):15299.
97. Zeleznik M, Cuk I, Pocrnjic M. Morphological Characteristics and Bilateral Differences of Youth U13, U15, U17 and U19 Male Football Players. International Journal of Morphology. 2023;41(1):257-63. doi: 10.4067/S0717-95022023000100257.
98. Zombra Ž. DIFFERENCES IN BODY COMPOSITION BETWEEN SOCCER PLAYERS AND NON-ATHLETES. Sport Scientific & Practical Aspects. 2019;16(1):47-52.
