# Supplementary material for: Bioelectrical Impedance Analysis in Professional and Semi-Professional Football: A Scoping Review
Source: Sports (Basel). 2025 Oct 3;13(10):348. doi: 10.3390/sports13100348 (PMC12568126; doi:10.3390/sports13100348)
Supplement: Supplementary file 1 [file sports-13-00348-s001.zip › sports-3865728-supplementary/sports-3865728-supplementary v5/Supplementary_4___Data_Charting.pdf]

Supplementary 4

Table S3 – Data charting of included studies

Authors and Citation	Year of Publication	Origin (country)	Primary Objectives	Population of Study	Reported BIA Outcomes and Measurement Time	BIA Device and Configuration	Key BIA Findings	General and Specific Applications/Research Areas
Bongiovanni et al. [1]	2020	Italy	Identify whether BIVA patters differ according to the competitive period in elite male players, and provide new BIVA references at the start of the season.	Italian first division male football players (*n* = 131; 25.1 ± 4.7 years old) (Statistic type unspecified).	PhA, R/H, Xc/H. End of preseason.	BIA-101 ®, Akern, Florence, Italy. Foot-to-hand. SF 50 kHz.	Raw bioelectrical parameters differ between two phases of the competition (end of preseason compared to previously published in-season values).	Qualitative or semi-quantitative body composition assessment: Raw bioelectrical parameters / sport phenotype assessment (cross-sectional RXc graph).<br><br>Longitudinal changes in raw bioelectrical parameters/dehydration-rehydration (PhA, R, Xc).
Bongiovanni et al. [2]	2021	Italy	Identify whether changes in regional PhA may act as a predictor of changes in vertical jump performance.	Italian first division male football players (*n* = 15; 28.7 ± 5.0 years old) (mean ± SD).	PhA (whole body and lower hemisome). Before preseason and after first the half of the season.	BIA-101 ®, Akern, Florence, Italy. Foot-to-hand. SF 50 kHz.	Changes in lower hemisome PhA are more strongly directly associated with changes in jump performance than changes in whole-body PhA.	Muscle health and function assessment: Comparisons of ICW/ECW & PhA and indicators of player's performance, external or internal load (e.g., GPS metrics, CMJ test scores, biomarkers of muscle damage).
Bongiovanni et al. [3]	2022	Italy	Examine the association between changes in body composition and hydration status with	Italian first division male football players (*n* = 22; 26.4 ± 4.8	ECW, ICW, PhA, R, TBW, Xc. Beginning and end of the	BIA-101 ®, Akern, Florence, Italy. Foot-to-hand. SF 50 kHz.	Changes in legs LST and ICW significantly explained improvements in CMJ height from the	Qualitative or semi-quantitative body composition assessment:

			changes in lower-body neuromuscular performance across a competitive season.	years old) (mean ± SD).	competitive season.		initial to the final stage of the competitive season.	Raw bioelectrical parameters / sport phenotype assessment (cross-sectional RXc graph) Longitudinal changes in raw bioelectrical parameters/dehydration-rehydration (PhA, R, Xc).<br><br>Muscle health and function assessment:<br>Comparisons of ICW/ECW & PhA and indicators of player's performance, external or internal load (e.g., GPS metrics, CMJ test scores, biomarkers of muscle damage).
Bongiovanni et al. [4]	2024	Italy	Describe body composition changes over the competitive season and compare the efficacy of different assessment methods to detect such changes.	Italian second division male football players (*n* = 21; 23.7 ± 4.8 years old) (Statistic type unspecified).	FFM, FM.<br>Four time points throughout the competitive season (October, December, February, April)	BIA-101 ®, Akern, Florence, Italy. Foot-to-hand. SF 50 kHz (for TBW estimation in the 4-component model)<br>and<br>Inbody 770 ®, Biospace, Seoul, South Korea. Foot-to-hand. MF-BIA.	While DXA and the 4-compartment model detected significant FFM increases—primarily in the lower limbs—during the season, most BIA methods showed limited sensitivity to these changes.	Quantitative body composition assessment:<br>Comparison with different methods for cross-sectional/longitudinal body composition assessment (e.g., FM, FFM).

Campa et al. [5]	2020	Italy	Propose new target zones for improving BIVA analysis in ball sports athletes.	Italian second division male football players (*n* = 41; 26.3 ± 3.2 years old) (mean ± SD).	ECW, FFM, FM, ICW, PhA, R/H, TBW, Xc/H Off-season.	BIA-101 ®, Akern, Florence, Italy. Foot-to-hand. SF 50 kHz.	A new target zone was proposed for male athletes in ball sports. BIVA patterns varied significantly according to somatotype. A higher endomorphic component was associated with a leftward shift in the RXc axis, while a greater mesomorphic component correlated with increased PhA.	Qualitative or semi-quantitative body composition assessment: Raw bioelectrical parameters / sport phenotype assessment (e.g., cross-sectional BIVA).
Campa et al. [6]	2020	Italy	Develop new equations for somatotype identification, integrating both anthropometry and BIA in elite male football players.	Italian first division male football players (*n* = 176; 26.9 ± 4.5 years old) (mean ± SD).	FFM, FM, PhA, R, Xc. Timing not specified.	BIA-101 ®, Akern, Florence, Italy. Foot-to-hand. SF 50 kHz.	A new model for somatotype assessment that accurately predicted somatotype components with high validity was proposed, with a reduced set of anthropometric inputs.	Quantitative body composition assessment: New predictive equation/model development.
Campa et al. [7]	2022	Italy	Examine whether menstrual cycle affects body composition and BIVA patterns, jumping and sprinting ability, and flexibility.	Italian first division female football players (*n* = 20; 23.8 ± 3.4 years old) (Statistic type unspecified).	FM, PhA, R/H, TBW, Xc/H. Off-season. Across two consecutive menstrual cycles.	BIA-101 ®, Akern, Florence, Italy. Foot-to-hand. SF 50 kHz.	The early follicular phase resulted in fluid accumulation, which was mirrored by BIVA vector shortening. No changes were observed in PhA.	Qualitative or semi-quantitative body composition assessment: Raw bioelectrical parameters / sport phenotype assessment (e.g., cross-sectional BIVA).<br><br>Longitudinal changes in raw bioelectrical parameters/dehydration-

								rehydration (PhA, R/H, Xc/H).
Campa et al. [8]	2022	Settings not specified	Develop reference PhA values for male and female athletes competing in various sport disciplines.	National level football players (male, *n* = 490; female, *n* = 55; age not specified).	PhA. In-season.	BIA-101 ®, Akern, Florence, Italy. Foot-to-hand. SF 50 kHz.	Normative PhA reference percentiles were provided for male and female football players. Male athletes consistently showed higher PhA than females, and endurance athletes had lower PhA than those in velocity/power or team sports.	Qualitative or semi-quantitative body composition assessment: Raw bioelectrical parameters / sport phenotype assessment (e.g., cross-sectional BIVA).
Campa et al. [9]	2023	Italy	Develop and validate a new BIA-based equation for estimating fat-free components (FFM, LST, ALST).	Italian first division male football players (*n* = 102; 24.7 ± 5.7 years old) (mean ± SD).	ALST, FFM, FM, LST, PhA, R, Xc. During the first half of the competitive season.	BIA-101 ®, Akern, Florence, Italy. Foot-to-hand. SF 50 kHz.	The new predictive equation was shown to provide more accurate estimations of body composition in elite football players compared to previous general equations developed for athletes.	Quantitative body composition assessment: New predictive equation/model development.
Fernandes et al. [10]	2022	Portugal	Evaluate whether intensity variations in external load influence BIA-derived body composition outcomes.	Portuguese first division female football players (*n* = 10; 24.6 ± 2.2 years old) (mean ± SD).	ECW, FFM, FM, ICW, PhA, TBW. Before and during the first half of the competitive season.	Inbody S10 ®, Biospace, Seoul, South Korea. Foot-to-hand. MF-BIA.	Only one external load variable (high-speed running) showed a significant correlation with one body composition variable (FM). No significant correlation between PhA and external load metrics was found.	Muscle health and function assessment: Comparisons of ICW/ECW & PhA and indicators of player's performance, external or internal load (e.g., GPS metrics, CMJ test scores, biomarkers of muscle damage).

Francavilla et al. [11]	2015	Italy	Assess whether localized BIA can detect and monitor physiological changes during the healing process of a muscle injury in a professional soccer player.	Italian first division male football player (*n* = 1; 24 years old).	PhA, R, Xc. --	BIA-101 ®, Akern, Florence, Italy. Localized. SF 50 kHz.	Localized BIA parameters (R, Xc, PhA) decreased significantly post-injury and progressively returned to baseline during recovery, reflecting fluid accumulation and cell membrane disruption.	Muscle health and function assessment: Localized BIA / muscle injury diagnosis and monitoring.
Gatterer et al. [12]	2011	Austria	Investigate the impact of repeated matches during the 2008 UEFA European Soccer Championship on hydration status and body composition in elite male.	National level male football players (*n* = 14; 25 ± years old) (mean ± SD).	BCM, ECM, FFM, FM, RXc graph, TBW. During the European Soccer Championship 2008.	BIA 2000-M ®, Data Input GmbH, Frankfurt, Germany. Foot-to-hand. MF-BIA.	Players competing in the European championship games experienced a decrease in extra-cellular mass and body weight. The impedance graph showed a vector lengthening, indicating fluid loss.	Quantitative body composition assessment: Monitoring hydration status (TBW).<br><br>Qualitative or semi-quantitative body composition assessment: Longitudinal changes in raw bioelectrical parameters/dehydration-rehydration (PhA, R/H, Xc/H).
Honorato et al. [13]	2023	Brazil	Assess the effects of a six-week preseason period on whole-body and regional BIA- and BIVA-derived parameters.	Professional male football players (*n* = 10; 30.0 ± 4.5 years old) (mean ± SD).	ECW, FFM, FM, ICW, PhA, R, TBW, Xc. Before, at mid-point and after a six-week preseason training period.	Quantum V Segmental BIA ®, RJL Systems, Clinton Township, MI, USA. Foot-to-hand. SF 50 kHz.	Only regional (hamstrings) phase angle increased significantly after pre-season, while whole-body phase angle remained unchanged.	Qualitative or semi-quantitative body composition assessment: Raw bioelectrical parameters / sport phenotype assessment (e.g., cross-sectional BIVA).<br><br>Longitudinal changes in raw bioelectrical parameters/dehydration-

								rehydration (PhA, R/H, Xc/H).
Leao et al. [14]	2017	Portugal	Evaluate differences between BIA, skinfold assessment and DXA in the estimation of body composition outcomes in elite young football players.	U19 National level male football players (*n* = 25; 17.28 ± 0.54 years old) (mean ± SD).	FM. In-season.	BC-418 ®, Tanita Corp., Tokyo, Japan. Foot-to-hand. SF-BIA.	BIA underestimates FM, whereas skinfolds showed a stronger correlation with DXA values.	Quantitative body composition assessment: Comparison with different methods for cross-sectional/longitudinal body composition assessment (i.e., FM, FFM).
Levi Micheli et al. [15]	2014	Italy	Establish bioelectrical impedance reference values for male soccer players across different competitive levels and evaluate whether BIVA and PhA can distinguish performance levels and differ from the general population.	Italian 1st-6th (professional and semi-professional) division male football players (*n* = 646; 26.1 ± 4.4 years old (1st-2nd), 24.5 ± 5.0 years old (3rd-4th), 22.2 ± 5.0 years old (5th-6th)) (mean ± SD).	Pha, R/H, Xc/H, TBW, FM, FFM, BCM, RXc graph. During the first half of the competitive season.	BIA-101 ®, Akern, Florence, Italy. Foot-to-hand. SF 50 kHz.	Muscle mass and function, as indicated by BCM and PhA, increase with higher performance level. Football-specific BIVA tolerance ellipses were proposed for performance classification and monitoring.	Qualitative or semi-quantitative body composition assessment: Raw bioelectrical parameters / sport phenotype assessment (e.g., cross-sectional BIVA).
Levi Micheli et al. [16]	2022	Italy	Propose a new BIVA derived parameter (LMI) that can allow a rapid assessment of the state of muscle mass in athletes, independent of predictive equations.	Italian 1st-4th division male football players (*n* = 664; 22.1 ± 5.1 – 26.4 ± 6.5 years old) (mean ± SD).	BCM, PhA, R, Xc. During the competitive season.	BIA-101 ®, Akern, Florence, Italy. Foot-to-hand. SF 50 kHz.	The proposed "Levi's Muscle Index" (LMI), consisting of expressing PhA adjusted by height and divided by R, was considered to provide more reliable information in the evaluation of muscle mass compared to traditional BIVA.	Qualitative or semi-quantitative body composition assessment: Raw bioelectrical parameters / sport phenotype assessment (e.g., cross-sectional BIVA).

Martinez-Ferran et al. [17]	2022	Spain	Compare anthropometric equations, BIA and absolute skinfold measurements using DXA as reference standard in the estimation of %FM.	Spanish first division male football players (*n* = 21; 26.3 ± 3.7 years old) (mean ± SD).	FM. During the first half of the competitive season.	BC-545N ®, Tanita Corp, Tokyo, Japan. Foot-to-hand. SF-BIA.	Strong correlations with DXA measurements were reported for all equations and separate skinfold measurements, yet only moderate correlations were observed for BIA, which underestimated %FM.	Quantitative body composition assessment: Comparison with different methods for cross-sectional/longitudinal body composition assessment (i.e., FM, FFM).
Mascherini et al. [18]	2015	Italy	Investigate whether changes in the bioelectrical impedance vector reflect performance changes throughout a competitive season.	Italian fourth division male football players (*n* = 18; 21.8 ± 3.0 years old) (mean ± SD).	BCM, ECW, FFM, FM, PhA, R, RXc graph, TBW, Xc During preseason and throughout the competitive season.	BIA-101 ®, Akern, Florence, Italy. Foot-to-hand. SF 50 kHz.	Vector changes consistent with body fluid gains were reported during preseason, which associated with enhanced endurance performance. Mid-season showed vector lengthening and increased phase angle (fluid loss and increased body cell mass), with no performance change. End-season showed fluid gain again.	Qualitative or semi-quantitative body composition assessment: Raw bioelectrical parameters / sport phenotype assessment (e.g., cross-sectional BIVA).<br><br>Longitudinal changes in raw bioelectrical parameters/dehydration-rehydration (PhA, R/H, Xc/H).
Mascherini et al. [19]	2015	Italy	Evaluate the impact of a 50-day physical training period on whole body, localized body composition outcomes and raw bioelectrical parameters.	Italian first division male football players (*n* = 59; 22.4 ± 5.5 years old) (mean ± SD).	BCM, ECW, FFM, FM, ICW, PhA, R/H, TBW, Xc/H. During preseason.	BIA-101 ®, Akern, Florence, Italy. Foot-to-hand and localized. SF 50 kHz.	BIA particularly when applied locally to specific muscle groups, is more sensitive than whole-body assessments in detecting training-induced changes in hydration and fat-free mass in elite soccer	Qualitative or semi-quantitative body composition assessment: Raw bioelectrical parameters / sport phenotype assessment (e.g., cross-sectional BIVA).<br><br>Longitudinal changes in raw bioelectrical

							players, especially in the lower limbs.	parameters/dehydration-rehydration (PhA, R/H, Xc/H).<br><br>Muscle health and function assessment:<br>Localized BIA / muscle injury diagnosis and monitoring.
Mascherini et al. [20]	2017	Italy	Compare male and female elite football players in terms of whole-body and localized body composition using a three-compartment model and localized bioimpedance analysis (BIA).	Italian first division male football players (*n* = 18; 26.2 ± 2.4 years old) and female football players (n = 18; 26.9 ± 2.5 years old) (mean ± SD).	BCM, ECW, FFM, FM, ICW, PhA, R/H, RXc graph, TBW, Xc/H<br>Before preseason.	BIA-101 ®, Akern, Florence, Italy. Foot-to-hand and localized. SF 50 kHz.	Localized bioimpedance analysis revealed significant sex-based differences in lower limb muscle composition, highlighting distinct segmental adaptations to training between sexes.	Qualitative or semi-quantitative body composition assessment:<br>Raw bioelectrical parameters / sport phenotype assessment (e.g., cross-sectional BIVA).<br><br>Muscle health and function assessment:<br>Localized BIA / muscle injury diagnosis and monitoring.
Mascherini et al. [21]	2019	Italy	Investigate sex differences in left ventricular mass in elite soccer players using body cell mass as a novel indexation method.	Italian first division male football players (*n* = 25; 26.5 ± 1.8 years old) and female football players (n = 25; 26.2 ± 1.9 years old) (mean ± SD).	BCM, ECM, FFM, FM, ICW, PhA, R, TBW, Xc<br>Timing not specified.	BIA-101 ®, Akern, Florence, Italy. Foot-to-hand. SF 50 kHz.	Body cell mass showed the strongest correlation with left ventricular mass, outperforming FFM and body surface are. Raw bioelectrical parameters were reported for both sexes.	Qualitative or semi-quantitative body composition assessment:<br>Raw bioelectrical parameters / sport phenotype assessment (e.g., cross-sectional BIVA)
Moya-Amaya et al. [22]	2021	Italy	To evaluate changes in PhA and muscle damage or inflammation markers 36 hours post-match, and	Italian first division male football players (*n* = 18; 26.4 ± 3.0	PhA.<br>First half of the competitive season.	MC-780MA ®, Tanita Corp., Tokyo, Japan.	PhA remained stable after the match but showed strong positive correlations with CK,	Muscle health and function assessment:<br>Comparisons of ICW/ECW & PhA and indicators of

			to determine the potential of phase angle as a post-match recovery monitoring marker.	years old) (mean ± SD).		Foot-to-hand. MF-BIA.	LDH, and CRP, suggesting its potential as a marker of muscle damage and inflammation during recovery.	player's performance, external or internal load (e.g., GPS metrics, CMJ test scores, biomarkers of muscle damage).
Munguia-Izquierdo et al. [23]	2018	Spain	Validate practical field methods for estimating %FM in elite male youth soccer players by comparing them to DXA, and develop a population-specific skinfold-based prediction equation.	Spanish elite male football players (n = 44; 17.1 ± 0.5 years old) (mean ± SD).	FM. During the competitive season.	Inbody 770 ®, Biospace, Seoul, South Korea. Foot-to-hand. MF-BIA. and MC-780MA ®, Tanita Corp., Tokyo, Japan. Foot to hand. MF-BIA.	BIA demonstrated a moderate correlation with DXA-derived FM values. This correlation was weaker than that observed with some anthropometric measurements.	Quantitative body composition assessment: Comparison with different methods for cross-sectional/longitudinal body composition assessment (i.e., FM, FFM).
Munguia-Izquierdo et al. [24]	2019	Spain	Determine the most effective field method for quantifying FFM in elite youth male soccer players compared to DXA.	Spanish elite male football players (n = 41; 17.1 ± 0.6 years old) (mean ± SD).	FFM. During the competitive season.	Inbody 770 ®, Biospace, Seoul, South Korea. Foot-to-hand. MF-BIA. and MC-780MA ®, Tanita Corp., Tokyo, Japan. Foot to hand. MF-BIA.	Several skinfold thickness equations were better predictors of DXA-derived FFM than BIA.	Quantitative body composition assessment: Comparison with different methods for cross-sectional/longitudinal body composition assessment (i.e., FM, FFM).
Nabuco et al. [25]	2019	Brazil	Examine the relationship between PhA and short-term maximal intensity efforts – specifically	Regional, national or international male football players (n = 99;	FM, FFM, PhA. Timing not specified.	Inbody S10 ®, Biospace, Seoul, South Korea.	PhA was inversely associated with fatigue index and positively related with maximum	Muscle health and function assessment: Comparisons of ICW/ECW & PhA and indicators of

			maximum power and fatigue index – in soccer players.	17.1 ± 0.6 years old) (mean ± SD).		Foot-to-hand. MF-BIA.	power, suggesting that PhA may be a reliable predictor of fatigue.	player's performance, external or internal load (e.g., GPS metrics, CMJ test scores, biomarkers of muscle damage).
Nescolarde et al. [26]	2013	Spain	Describe relative changes in BIA and vector data relative to the severity of injuries in selected muscle groups.	Spanish first division male football players (*n* = 3; 3 muscle injuries; 19-26 years old) (range).	PhA, R, RXc graph, Xc. --	BIA-101 ®, Akern, Florence, Italy. Localized. SF 50 kHz.	Compared to non-injury values, R, Xc and PhA decreased with increasing muscle injury severity. R reflected fluid accumulation, while Xc and PA indicated cellular membrane disruption. These parameters progressively returned toward baseline during recovery.	Muscle health and function assessment: Localized BIA / muscle injury diagnosis and monitoring.
Nescolarde et al. [27]	2014	Spain	Determine changes in BIA components, 24 h after injury, in a group of muscle injuries depending on their severity as confirmed by MRI.	Spanish first division male football players (sample size not reported; 21 muscle injuries; age not specified).	PhA, R, Xc. --	BIA-101 ®, Akern, Florence, Italy. Localized. SF 50 kHz.	The most significant changes 24 h after injury was the sizeable decrease in Xc that indicates a pattern of disrupted soft tissue structure, proportional to the severity of the injury.	Muscle health and function assessment: Localized BIA / muscle injury diagnosis and monitoring.
Nescolarde et al. [28]	2017	Spain	Evaluate whether L-BIA can detect muscle gaps (fiber retraction) in muscle injuries and predict RTP time.	Spanish first division male football players (*n* = 18; 22 muscle injuries; 20-28 years old) (range).	PhA, R, Xc. --	BIA-101 ®, Akern, Florence, Italy. Localized. SF 50 kHz.	Xc and PA values decreased significantly with injury severity and were sensible to the presence of muscle gap. L-BIA could aid MRI and ultrasound in injury	Muscle health and function assessment: Localized BIA / muscle injury diagnosis and monitoring.

							severity assessment and RTP time prediction.	
Nescolarde et al. [29]	2020	Spain	Determine whether L-BIA can differentiate between tendinous, MTJ, and MFJ injuries in professional football players 24 hours post-injury (as diagnosed by MRI); to assess the severity of MTJ injuries (grades 1–3) using L-BIA; and to explore the relationship between L-BIA parameters and RTP time.	Spanish first division male football players (*n* = 32; 37 muscle injuries; 20-30 years old) (range).	PhA, R, Xc.<br>--	BIA-101 ®, Akern, Florence, Italy. Localized. SF 50 kHz.	L-BIA parameters significantly decreased in MTJ and MFJ injuries compared to the non-injured leg, particularly Xc, which is associated with muscle cell disruption. No significant differences were observed in tendinous injuries. Greater severity of MTJ injuries corresponded with larger reductions in Xc and longer RTP duration.	Muscle health and function assessment:<br>Localized BIA / muscle injury diagnosis and monitoring.
Núñez et al. [30]	2020	Spain	Determine which field methods (anthropometric equations or BIA) are most effective in detecting FFM changes in elite youth soccer players across the season, using DXA as reference.	Spanish elite male football players (*n* = 40; 16.6 ± 0.5 years old) (mean ± SD).	FFM.<br>Preseason and mid-season.	Inbody 770 ®, Biospace, Seoul, South Korea. Foot-to-hand. MF-BIA.<br>and<br>BC-418 ®, Tanita Corp., Tokyo, Japan. Foot to hand. SF-BIA.	Specific anthropometric equations were better predictors of DXA-derived FFM than BIA.	Quantitative body composition assessment:<br>Comparison with different methods for cross-sectional/longitudinal body composition assessment (i.e., FM, FFM).
Oliveira et al. [31]	2021	Portugal	Analyze variations in body composition variables and their relationship with	Portuguese first division female football players (*n* = 17; 22.7 ± 6.3	ECW, FFM, FM, ICW, PhA, TBW.<br>During the competitive season.	Inbody S10 ®, Biospace, Seoul, South Korea. Foot-to-hand. MF-BIA.	No linear interaction was demonstrated between internal load (RPE) and any BIA outcomes, including PhA.	Muscle health and function assessment:<br>Comparisons of ICW/ECW & PhA and indicators of player's performance,

			internal load (RPE) using BIA.	years old) (mean ± SD).				external or internal load (e.g., GPS metrics, CMJ test scores, biomarkers of muscle damage).
Oliveira et al. [32]	2023	Portugal	Compare physical, physiological, body composition (BIA), and load markers between starters and non-starters.	Portuguese first division female football players (*n* = 14; 23.2 ± 3.1 years old) (mean ± SD).	ECW, FFM, FM, ICW, PhA, TBW. Before and after a five-week preseason.	Inbody S10 ®, Biospace, Seoul, South Korea. Foot-to-hand. MF-BIA.	Several correlations were observed between body composition variables, most notably PhA, and performance metrics (VO_2max, CMJ).	Muscle health and function assessment: Comparisons of ICW/ECW & PhA and indicators of player's performance, external or internal load (e.g., GPS metrics, CMJ test scores, biomarkers of muscle damage).
Petri et al. [33]	2016	Italy	Assess dietary habits and body composition (BIA) outcomes of elite male soccer players over the course of a competitive season.	Italian first division male football players (*n* = 28; 27.8 ± 4.5 years old) (mean ± SD).	PhA, R, Xc. Before and after preseason, and at the end of the competitive season.	BIA-101 ®, Akern, Florence, Italy. Foot-to-hand. SF 50 kHz.	Raw bioelectric parameters did not significantly change across pre-season, start of season and end of season.	Qualitative or semi-quantitative body composition assessment: Raw bioelectrical parameters / sport phenotype assessment (e.g., cross-sectional BIVA).<br><br>Longitudinal changes in raw bioelectrical parameters/dehydration-rehydration (PhA, R/H, Xc/H).
Petri et al. [34]	2024	Italy	Evaluate the effects of a structured nutritional program led by a sport nutritionist on body composition outcomes, using anthropometry and BIA across two	Italian first division male football players (*n* = 44; 26.2 ± 2.9 years old) and female football players (n = 44; 27	BCM, FM, PhA, R, Xc. End of the competitive season.	BIA-101 ®, Akern, Florence, Italy. Foot-to-hand. SF 50 kHz.	Across both sexes, BIA underestimated FM and showed limited sensitivity to changes compared to skinfold-based assessments.	Quantitative body composition assessment: Comparison with different methods for cross-sectional/longitudinal body composition assessment (i.e., FM, FFM).

			seasons (2014/15 and 2022/23).	± 5.0 years old) (mean ± SD).				Qualitative or semi-quantitative body composition assessment: Raw bioelectrical parameters / sport phenotype assessment (e.g., cross-sectional BIVA).
Ramirez-Munera et al. [35]	2024	Spain	Identify position-specific changes in BIA-derived body composition estimates during preseason and examine associations with different anthropometric equations.	Spanish first and second division female football players (*n* = 34; 23.0 ± 4.2 years old) (mean ± SD).	Bone mass, FM, FFM, TBW. Before and after preseason.	BC-420 S MA Master Class III ®, Tanita Corp., Tokyo, Japan. Foot-to-hand. SF-BIA.	A decreased in FM was observed, particularly in midfielders. There was a significant correlation between Faulkner's equation and BIA in terms of %FM. However, BIA tended to underestimate fat-free mass and muscle mass.	Quantitative body composition assessment: Comparison with different methods for cross-sectional/longitudinal body composition assessment (i.e., FM, FFM).
Suarez-Arrones et al. [36]	2018	Italy	Cross-validate three field methods (BIA, skinfold thickness equations, and skinfold sums) for quantifying FM against DXA.	Italian first division male soccer players (*n* = 18; 27.6 ± 3.0 years old) (mean ± SD).	FM. End of the competitive season.	MC-180 MA III ®, Tanita Corp., Tokyo, Japan. Foot-to-hand. MF-BIA.	Anthropometric equations were better predictors of DXA derived FM than BIA. Specifically, BIA consistently underestimated %FM compared to DXA.	Quantitative body composition assessment: Comparison with different methods for cross-sectional/longitudinal body composition assessment (i.e., FM, FFM).
Svantesson et al. [37]	2008	Sweden	Compare body composition results obtained with BIS and DXA in male athletes.	Swedish first division male soccer players (*n* = 17; 24.1 ± 3.8 years old) (mean ± SD).	FFM, FM. Timing not specified.	Hydra 4200 ®, Xitron Technologies Inc., San Diego, CA, USA. Foot-to-hand. BIS.	Lower values of FM were observed with BIS in comparison with DXA.	Quantitative body composition assessment: Comparison with different methods for cross-sectional/longitudinal body composition assessment (i.e., FM, FFM).

Tornero-Aguilera et al. [38]	2022	Spain	Compare %FM estimated by anthropometric measurements (skinfold thickness) and BIA methods to that reported by DXA.	National-level male (*n* = 70; 21.8 ± 5.0 years old) and female football players (*n* = 76; 22.2 ± 3.2 years old) (mean ± SD).	FM. Before the preseason.	Inbody 770 ®, Biospace, Seoul, South Korea. Foot-to-hand. MF-BIA.	BIA significantly underestimated %FM in comparison with DXA, while skinfold thickness measurements also underestimated it, but to a lesser extent.	Quantitative body composition assessment: Comparison with different methods for cross-sectional/longitudinal body composition assessment (i.e., FM, FFM).
Yargic et al. [39]	2020	Turkey	Evaluate seasonal alterations in body composition and compare differences between skinfold equations and BIA.	Turkish first division male soccer players (*n* = 24; 26.3 ± 4.5 years old) (mean ± SD).	Bone mass, FM, TBW. At preseason and during the competitive season.	MC-980MA ®, Tanita Corp., Tokyo, Japan. Foot-to-hand. MF-BIA.	No seasonal changes in any body composition parameter were observed. Differences were observed between methods.	Quantitative body composition assessment: Comparison with different methods for cross-sectional/longitudinal body composition assessment (i.e., FM, FFM).

ALST, appendicular lean soft tissue; BCM, body cell mass; BIA, bioelectrical impedance analysis; BIS, bioelectrical impedance spectroscopy; BIVA, bioelectrical impedance vector analysis; CMJ, countermovement jump; CRP, C-reactive protein; DXA, dual-energy X-ray absorptiometry; ECM, extracellular mass; ECW, extracellular water; FFM, fat-free mass; FM, fat mass; GPS, global positioning system; H, height; ICW, intracellular water; LMI, Levi's muscle index; LST, lean soft tissue; MF-BIA, multi-frequency bioelectrical impedance analysis; MRI, magnetic resonance imaging; MTJ, myotendinous junction; PhA, phase angle; R, resistance; R/H, resistance normalized by height; RPE, rating of perceived exertion; RXc graph, resistance–reactance graph; RTP, return to play; SD, standard deviation; SF, single frequency; TBW, total body water; VO_2max, maximal oxygen uptake; Xc, reactance; Xc/H, reactance normalized by height.

References

1. Bongiovanni, T.; Mascherini, G.; Genovesi, F.; Pasta, G.; Iaia, F.M.; Trecroci, A.; Ventimiglia, M.; Alberti, G.; Campa, F. Bioimpedance Vector References Need to Be Period-Specific for Assessing Body Composition and Cellular Health in Elite Soccer Players: A Brief Report. *Journal of Functional Morphology and Kinesiology* **2020**, *5*, 73.

2. Bongiovanni, T.; Trecroci, A.; Rossi, A.; Iaia, F.M.; Pasta, G.; Campa, F. Association between change in regional phase angle and jump performance: A pilot study in serie a soccer players. *European Journal of Investigation in Health, Psychology and Education* **2021**, *11*, 860-865.
3. Bongiovanni, T.; Rossi, A.; Trecroci, A.; Martera, G.; Iaia, F.M.; Alberti, G.; Pasta, G.; Lacome, M. Regional bioelectrical phase angle is more informative than whole-body phase angle for monitoring neuromuscular performance: a pilot study in elite young soccer players. *Sports* **2022**, *10*, 66.
4. Bongiovanni, T.; Lacome, M.; Rodriguez, C.; Tinsley, G.M. Tracking Body Composition Over a Competitive Season in Elite Soccer Players Using Laboratory- and Field-Based Assessment Methods. *Journal of strength and conditioning research* **2024**, *38*, e104-e115, doi:10.1519/jsc.0000000000004662.
5. Campa, F.; Bongiovanni, T.; Matias, C.N.; Genovesi, F.; Trecroci, A.; Rossi, A.; Iaia, F.M.; Alberti, G.; Pasta, G.; Toselli, S. A New Strategy to Integrate Heath–Carter Somatotype Assessment with Bioelectrical Impedance Analysis in Elite Soccer Players. *Sports* **2020**, *8*, 142.
6. Campa, F.; Silva, A.M.; Talluri, J.; Matias, C.N.; Badicu, G.; Toselli, S. Somatotype and Bioimpedance Vector Analysis: A New Target Zone for Male Athletes. *Sustainability* **2020**, *12*, 4365.
7. Campa, F.; Levi Micheli, M.; Pompignoli, M.; Cannataro, R.; Gulisano, M.; Toselli, S.; Greco, G.; Coratella, G. The Influence of Menstrual Cycle on Bioimpedance Vector Patterns, Performance, and Flexibility in Elite Soccer Players. *Int J Sports Physiol Perform* **2022**, *17*, 58-66, doi:10.1123/ijspp.2021-0135.
8. Campa, F.; Thomas, D.M.; Watts, K.; Clark, N.; Baller, D.; Morin, T.; Toselli, S.; Koury, J.C.; Melchiorri, G.; Andreoli, A.; et al. Reference Percentiles for Bioelectrical Phase Angle in Athletes. *Biology* **2022**, *11*, 264.
9. Campa, F.; Bongiovanni, T.; Rossi, A.; Cerullo, G.; Casolo, A.; Martera, G.; Trecroci, A.; Moro, T.; Paoli, A. Athletic bioimpedance-based equations underestimate fat free mass components in male elite soccer players: development and validation of new soccer-specific predictive models. *Journal of Translational Medicine* **2023**, *21*, 912, doi:10.1186/s12967-023-04795-z.
10. Fernandes, R.; Martins, A.D.; Clemente, F.M.; Brito, J.P.; Nobari, H.; Reis, V.; Oliveira, R. Variations of distance and accelerometry-based GPS measures and their influence on body composition in professional women soccer players. *Proceedings of the Institution of Mechanical Engineers, Part P: Journal of Sports Engineering and Technology* **2025**, *239*, 20-28, doi:10.1177/17543371221122076.
11. Francavilla, V.; Bongiovanni, T.; Genovesi, F.; Minafra, P.; Francavilla, G. Localized bioelectrical impedance analysis: How useful is it in the follow-up of muscle injury? A case report. *Medicina dello sport; rivista di fisiopatologia dello sport* **2015**, *68*, 323-334.
12. Gatterer, H.; Schenk, K.; Ferrari, P.; Faulhaber, M.; Schopp, E.; Burtscher, M. Changes in hydration status of soccer players competing in the 2008 European Championship. *The Journal of sports medicine and physical fitness* **2011**, *51*, 89-94.

13. Honorato, R.d.C.; Soares Marreiros Ferraz, A.; Kassiano, W.; Martins, P.C.; Silva, D.A.S.; Ceccatto, V.M. Regional phase angle, not whole-body, is augmented in response to pre-season in professional soccer players. *Research in Sports Medicine* **2023**, *31*, 831-845, doi:10.1080/15438627.2022.2052069.
14. Leão, C.; Simões, M.; Silva, B.; Clemente, F.M.; Bezerra, P.; Camões, M. Body Composition Evaluation Issue among Young Elite Football Players: DXA Assessment. *Sports (Basel, Switzerland)* **2017**, *5*, doi:10.3390/sports5010017.
15. Micheli, M.L.; Pagani, L.; Marella, M.; Gulisano, M.; Piccoli, A.; Angelini, F.; Burtscher, M.; Gatterer, H. Bioimpedance and impedance vector patterns as predictors of league level in male soccer players. *International journal of sports physiology and performance* **2014**, *9*, 532-539.
16. Levi Micheli, M.; Cannataro, R.; Gulisano, M.; Mascherini, G. Proposal of a New Parameter for Evaluating Muscle Mass in Footballers through Bioimpedance Analysis. *Biology* **2022**, *11*, doi:10.3390/biology11081182.
17. Martinez-Ferran, M.; Rafei, E.; Romero-Morales, C.; Pérez-Ruiz, M.; Lam-Meléndez, A.; Munguia-Izquierdo, D.; Pareja-Galeano, H. Optimizing Field Body Fat Percentage Assessment in Professional Soccer Players. *Applied Sciences* **2022**, *12*, 727.
18. Mascherini, G.; Gatterer, H.; Lukaski, H.; Burtscher, M.; Galanti, G. Changes in hydration, body-cell mass and endurance performance of professional soccer players through a competitive season. *The Journal of sports medicine and physical fitness* **2015**, *55*, 749-755.
19. Mascherini, G.; Petri, C.; Galanti, G. Integrated total body composition and localized fat-free mass assessment. *Sport Sciences for Health* **2015**, *11*, 217-225, doi:10.1007/s11332-015-0228-y.
20. Mascherini, G.; Castizo-Olier, J.; Irurtia, A.; Petri, C.; Galanti, G. Differences between the sexes in athletes' body composition and lower limb bioimpedance values. *Muscles, ligaments and tendons journal* **2017**, *7*, 573-581, doi:10.11138/mltj/2017.7.4.573.
21. Mascherini, G.; Petri, C.; Galanti, G. Link between body cellular mass and left ventricular hypertrophy in female and male athletes. *The Journal of sports medicine and physical fitness* **2019**, *59*, 164-170, doi:10.23736/s0022-4707.18.08259-2.
22. Moya-Amaya, H.; Molina-López, A.; Berralaguilar, A.J.; Rojano-Ortega, D.; Berral-De La Rosa, C.J.; Berral-De La Rosa, F.J. Bioelectrical phase angle, muscle damage markers and inflammatory response after a competitive match in professional soccer players. *Polish Journal of Sport and Tourism* **2021**, *28*, 8-13.
23. Munguia-Izquierdo, D.; Suarez-Arrones, L.; Di Salvo, V.; Paredes-Hernandez, V.; Alcazar, J.; Ara, I.; Kreider, R.; Mendez-Villanueva, A. Validation of field methods to assess body fat percentage in elite youth soccer players. *International journal of sports medicine* **2018**, *39*, 349-354.

24. Munguía-Izquierdo, D.; Suárez-Arrones, L.; Di Salvo, V.; Paredes-Hernández, V.; Ara, I.; Mendez-Villanueva, A. Estimating fat-free mass in elite youth male soccer players: cross-validation of different field methods and development of prediction equation. *Journal of Sports Sciences* **2019**, *37*, 1197-1204.
25. Nabuco, H.C.; Silva, A.M.; Sardinha, L.B.; Rodrigues, F.B.; Tomeleri, C.M.; Ravagnani, F.C.; Cyrino, E.S.; Ravagnani, C.F. Phase angle is moderately associated with short-term maximal intensity efforts in soccer players. *International journal of sports medicine* **2019**, *40*, 739-743.
26. Nescolarde, L.; Yanguas, J.; Lukaski, H.; Alomar, X.; Rosell-Ferrer, J.; Rodas, G. Localized bioimpedance to assess muscle injury. *Physiological measurement* **2013**, *34*, 237.
27. Nescolarde, L.; Yanguas, J.; Lukaski, H.; Alomar, X.; Rosell-Ferrer, J.; Rodas, G. Effects of muscle injury severity on localized bioimpedance measurements. *Physiological measurement* **2014**, *36*, 27.
28. Nescolarde, L.; Yanguas, J.; Terricabras, J.; Lukaski, H.; Alomar, X.; Rosell-Ferrer, J.; Rodas, G. Detection of muscle gap by L-BIA in muscle injuries: clinical prognosis. *Physiological measurement* **2017**, *38*, L1.
29. Nescolarde, L.; Terricabras, J.; Mechó, S.; Rodas, G.; Yanguas, J. Differentiation Between Tendinous, Myotendinous and Myofascial Injuries by L-BIA in Professional Football Players. *Front Physiol* **2020**, *11*, 574124, doi:10.3389/fphys.2020.574124.
30. Núñez, F.J.; Munguía-Izquierdo, D.; Suárez-Arrones, L. Validity of Field Methods to Estimate Fat-Free Mass Changes Throughout the Season in Elite Youth Soccer Players. *Frontiers in Physiology* **2020**, *Volume 11 - 2020*, doi:10.3389/fphys.2020.00016.
31. Oliveira, R.; Francisco, R.; Fernandes, R.; Martins, A.; Nobari, H.; Clemente, F.M.; Brito, J.P. In-Season Body Composition Effects in Professional Women Soccer Players. *Int J Environ Res Public Health* **2021**, *18*, doi:10.3390/ijerph182212023.
32. Oliveira, R.; Brito, J.P.; Fernandes, R.; Morgans, R.; Alves, S.; Santos, F.J.; Pinto, P.; Espada, M.C. The Effects of Pre-Season and Relationships with Physical, Physiological, Body Composition, and Load Markers: A Case Study Comparing Starters versus Non-Starters from an Elite Female Professional Soccer Team. *Medicina (Kaunas, Lithuania)* **2023**, *59*, doi:10.3390/medicina59122156.
33. Petri, C.; Mascherini, G.; Pengue, L.; Galanti, G. Dietary habits in elite soccer players. *Sport Sciences for Health* **2016**, *12*, 113-119, doi:10.1007/s11332-016-0264-2.
34. Petri, C.; Pengue, L.; Bartolini, A.; Pistolesi, D.; Arrones, L.S. Body Composition Changes in Male and Female Elite Soccer Players: Effects of a Nutritional Program Led by a Sport Nutritionist. *Nutrients* **2024**, *16*, doi:10.3390/nu16030334.
35. Ramírez-Munera, M.; Arcusa, R.; López-Román, F.J.; Victoria-Montesinos, D.; García-Muñoz, A.M.; Ávila-Gandía, V.; Pérez-Piñero, S.; Marhuenda, J. Anthropometric and Body Composition Changes during Pre-Season of Spanish Professional Female Soccer Players According to Playing Position. *Nutrients* **2024**, *16*, 2799.

36. Suarez-Arrones, L.; Petri, C.; Maldonado, R.A.; Torreno, N.; Munguía-Izquierdo, D.; Di Salvo, V.; Méndez-Villanueva, A. Body fat assessment in elite soccer players: cross-validation of different field methods. *Science and Medicine in Football* **2018**, *2*, 203-208.
37. Svantesson, U.; Zander, M.; Klingberg, S.; Slinde, F. Body composition in male elite athletes, comparison of bioelectrical impedance spectroscopy with dual energy X-ray absorptiometry. *Journal of negative results in biomedicine* **2008**, *7*, 1, doi:10.1186/1477-5751-7-1.
38. Tornero-Aguilera, J.F.; Villegas-Mora, B.E.; Clemente-Suárez, V.J. Differences in Body Composition Analysis by DEXA, Skinfold and BIA Methods in Young Football Players. *Children (Basel, Switzerland)* **2022**, *9*, doi:10.3390/children9111643.
39. Yargic, M.P.; Kurklu, G.B.; Celen, M.C.; Goktepe, E. Seasonal body composition alterations of an elite male soccer team evaluated with skinfold thickness equations and BIMP analysis. *COMPARATIVE EXERCISE PHYSIOLOGY* **2020**, *16*, 339-346, doi:10.3920/CEP200004.
