# Supplementary material for: Bioelectrical Impedance Analysis in Professional and Semi-Professional Football: A Scoping Review
Source: Sports (Basel). 2025 Oct 3;13(10):348. doi: 10.3390/sports13100348 (PMC12568126; doi:10.3390/sports13100348)
Supplement: Supplementary file 1 [file sports-13-00348-s001.zip › sports-3865728-supplementary/sports-3865728-supplementary v5/Supplementary_5___BIVA_Data_Extraction.pdf]

Table S4 – Data extraction of studies reporting raw bioelectrical parameters

Authors and Citation	Year of Publication	Population of Study	Measurement Time	R (Ohm)	Xc (Ohm)	H (m)	W (kg)	PhA (º)
Bongiovanni et al. [1]	2020	Italian first division male football players (*n* = 131; 25.1 ± 4.7 years old) (Statistic type unspecified)	End of preseason.	451.04	62.9	1.818	79.3	8.0
Bongiovanni et al. [2]	2022	Italian first division male football players (*n* = 22; 26.4 ± 4.8 years old) (mean ± SD)	Beginning (T0) and end of the competitive season (T1)	465.27 (T0)<br>467.09 (T1)	65.09 (T0)<br>66.09 (T1)	1.843	81.2 (T0)<br>82.5 (T1)	8.01 (T0)<br>8.11 (T1)
Campa et al. [3]	2020	Italian second division male football players (*n* = 41; 26.3 ± 3.2 years old) (mean ± SD)	Off-season	461.61	63.75	1.806	74.7	7.9
Honorato et al. [4]	2023	Professional male football players (*n* = 10; 30.0 ± 4.5 years old) (mean ± SD).	Before (T0), during (T1), and after preseason (T2)	402.8 (T0)<br>375.6 (T1)<br>366.0 (T2)	76.7 (T0)<br>71.1 (T1)<br>74.5 (T2)	1.80	81.5 (T0)<br>81.8 (T1)<br>82.1 (T2)	10.9 (T0)<br>10.8 (T1)<br>11.7 (T2)
Levi Micheli et al. [5]	2014	Italian 1st-6th (professional and semi-professional) division male football players (*n* = 646; 26.1 ± 4.4 years old (1st-2nd), 24.5 ± 5.0 years old (3rd-4th), 22.2 ± 5.0 years old (5th-6th)) (mean ± SD).	First half of the competitive season	458.56 (1st-2nd)<br>469.56 (3rd-4th)<br>486.27 (5th-6th)	61.66 (1st-2nd)<br>60.86 (3rd-4th)<br>60.74 (5th-6th)	1.819 (1st-2nd)<br>1.806 (3rd-4th)<br>1.797 (5th-6th)	79.2 (1st-2nd)<br>75.8 (3rd-4th)<br>73.9 (5th-6th)	7.7 (1st-2nd)<br>7.4 (3rd-4th)<br>7.1 (5th-6th)
Mascherini et al.[6]	2015	Italian fourth division male football players (*n* = 18; 21.8 ± 3.0 years old) (mean ± SD).	Preseason (T1-T3), mid-season (T4-T7), and end-of-season (T8)	490.14 (T0)<br>472.12 (T1)<br>475.73 (T2)<br>501.50 (T3)<br>493.93 (T4)<br>496.27 (T5)<br>492.49 (T6)<br>478.43 (T7)	64.87 (T0)<br>61.09 (T1)<br>60.91 (T2)<br>70.64 (T3)<br>68.12 (T4)<br>71.18 (T5)<br>69.20 (T6)<br>67.39 (T7)	1.802	74.2 (T0)<br>74.6 (T1)<br>74.2 (T2)<br>74.4 (T3)<br>75.4 (T4)<br>75.2 (T5)<br>75.0 (T6)<br>75.0 (T7)	7.5 (T0)<br>7.4 (T1)<br>7.3 (T2)<br>8.0 (T3)<br>7.9 (T4)<br>8.2 (T5)<br>8.0 (T6)<br>8.0 (T7)
Mascherini et al.[7]	2015	Italian first division male football players (*n* = 59; 22.4 ± 5.5 years old) (mean ± SD).	Before (T0) and after preseason (T1)	470.29 (T0)<br>464.46 (T1)	64.18 (T0)<br>62.97 (T1)	1.81	76.48 (T0)<br>77.11 (T1)	7.80<br>7.73

Mascherini et al. [8]	2017	Italian first division male football players (*n* = 18; 26.2 ± 2.4 years old) and female football players (n = 18; 26.9 ± 2.5 years old) (mean ± SD).	Before preseason	463.15	64.32	1.822	77.9	7.9
Mascherini et al. [9]	2019	Italian first division male football players (*n* = 25; 26.5 ± 1.8 years old) and female football players (*n* = 25; 26.2 ± 1.9 years old) (mean ± SD).	Timing not specified	467.92	64.79	1.82	77.6	7.8
Petri et al. [10]	2016	Italian first division male football players (*n* = 28; 27.8 ± 4.5 years old) (mean ± SD).	Before preseason (T0), end of preseason (T1), and end-of-season (T2).	462.39 (T0) 462.58 (T1) 465.34 (T2)	64.40 (T0) 62.56 (T1) 62.19 (T2)	1.84	79.9 (T0) 81.4 (T1) 78.6 (T2)	7.9 (T0) 7.7 (T1) 7.6 (T2)
Petri et al. [11]	2024	Italian first division male football players (*n* = 44; 26.2 ± 2.9 years old) and female football players (*n* = 44; 27 ± 5.0 years old) (mean ± SD).	In-season	436.0	65.8	1.84	80.9	8.6

Mean values are presented. H, height; PhA, phase angle; SD, standard deviation; R, resistance; T, time point; W, weight; Xc, reactance

References

1. Bongiovanni, T.; Mascherini, G.; Genovesi, F.; Pasta, G.; Iaia, F. M.; Trecroci, A.; Ventimiglia, M.; Alberti, G.; Campa, F., Bioimpedance Vector References Need to Be Period-Specific for Assessing Body Composition and Cellular Health in Elite Soccer Players: A Brief Report. *Journal of Functional Morphology and Kinesiology* **2020,** *5* (4), 73.
2. Bongiovanni, T.; Rossi, A.; Trecroci, A.; Martera, G.; Iaia, F. M.; Alberti, G.; Pasta, G.; Lacome, M., Regional bioelectrical phase angle is more informative than whole-body phase angle for monitoring neuromuscular performance: a pilot study in elite young soccer players. *Sports* **2022,** *10* (5), 66.
3. Campa, F.; Bongiovanni, T.; Matias, C. N.; Genovesi, F.; Trecroci, A.; Rossi, A.; Iaia, F. M.; Alberti, G.; Pasta, G.; Toselli, S., A New Strategy to Integrate Heath–Carter Somatotype Assessment with Bioelectrical Impedance Analysis in Elite Soccer Players. *Sports* **2020,** *8* (11), 142.
4. Honorato, R. d. C.; Soares Marreiros Ferraz, A.; Kassiano, W.; Martins, P. C.; Silva, D. A. S.; Ceccatto, V. M., Regional phase angle, not whole-body, is augmented in response to pre-season in professional soccer players. *Research in Sports Medicine* **2023,** *31* (6), 831-845.
5. Micheli, M. L.; Pagani, L.; Marella, M.; Gulisano, M.; Piccoli, A.; Angelini, F.; Burtscher, M.; Gatterer, H., Bioimpedance and impedance vector patterns as predictors of league level in male soccer players. *International journal of sports physiology and performance* **2014,** *9* (3), 532-539.
6. Mascherini, G.; Gatterer, H.; Lukaski, H.; Burtscher, M.; Galanti, G., Changes in hydration, body-cell mass and endurance performance of professional soccer players through a competitive season. *The Journal of sports medicine and physical fitness* **2015,** *55* (7-8), 749-55.
7. Mascherini, G.; Petri, C.; Galanti, G., Integrated total body composition and localized fat-free mass assessment. *Sport Sciences for Health* **2015,** *11* (2), 217-225.
8. Mascherini, G.; Castizo-Olier, J.; Irurtia, A.; Petri, C.; Galanti, G., Differences between the sexes in athletes' body composition and lower limb bioimpedance values. *Muscles, ligaments and tendons journal* **2017,** *7* (4), 573-581.
9. Mascherini, G.; Petri, C.; Galanti, G., Link between body cellular mass and left ventricular hypertrophy in female and male athletes. *The Journal of sports medicine and physical fitness* **2019,** *59* (1), 164-170.
10. Petri, C.; Mascherini, G.; Pengue, L.; Galanti, G., Dietary habits in elite soccer players. *Sport Sciences for Health* **2016,** *12* (1), 113-119.
11. Petri, C.; Pengue, L.; Bartolini, A.; Pistolesi, D.; Arrones, L. S., Body Composition Changes in Male and Female Elite Soccer Players: Effects of a Nutritional Program Led by a Sport Nutritionist. *Nutrients* **2024,** *16* (3).
